# A Rotational Pressure-Correction Scheme for Incompressible Two-Phase Flows with Open Boundaries

**DOI:** 10.1371/journal.pone.0154565

**Published:** 2016-05-10

**Authors:** S. Dong, X. Wang

**Affiliations:** Center for Computational & Applied Mathematics, Department of Mathematics, Purdue University, West Lafayette, Indiana, United States of America; Tianjin University, CHINA

## Abstract

Two-phase outflows refer to situations where the interface formed between two immiscible incompressible fluids passes through open portions of the domain boundary. We present several new forms of open boundary conditions for two-phase outflow simulations within the phase field framework, as well as a rotational pressure correction based algorithm for numerically treating these open boundary conditions. Our algorithm gives rise to linear algebraic systems for the velocity and the pressure that involve only constant and time-independent coefficient matrices after discretization, despite the variable density and variable viscosity of the two-phase mixture. By comparing simulation results with theory and the experimental data, we show that the method produces physically accurate results. We also present numerical experiments to demonstrate the long-term stability of the method in situations where large density contrast, large viscosity contrast, and backflows occur at the two-phase open boundaries.

## Introduction

The current work focuses on the motion of a mixture of two immiscible incompressible fluids in a domain that is open on part of its boundary. The domain boundary is open in the sense that the fluids can freely leave or even enter the domain through such boundaries. In particular, we assume that the interface formed between the two fluids will pass through the open portions of the domain boundary. Therefore, the problem will involve truly two-phase outflow/open boundaries.

Two-phase outflows are encountered in many situations: oil plumes in the deep sea, wakes of surface ships, and ocean waves generated by the wind shear are some examples. The slug/churn/bubbly flows crucial in many industrial processes (see e.g. the experiments of [1–3] for their dynamical characterizations) provide other examples of two-phase flow situations of this type. These problems usually involve physically unbounded flow domains. Numerical simulation of such problems will therefore need to truncate the domain to a finite size, and some open/outflow boundary condition (OBC) will be required on the artificial boundary. The presence of fluid interfaces at the open boundary calls for appropriate two-phase open boundary conditions in such problems.

Several challenges are associated with the design of two-phase open boundary conditions. Some of the challenges are common to those encountered with single-phase outflows, for example, the instability associated with strong vortices or backflows at the open boundary (a.k.a. backflow instability) [4, 5]. Others are new and unique to two-phase outflows. For example, owing to the presence of fluid interfaces, two-phase outflow problems involve viscosity contrasts, density contrasts, and surface tension at the open boundaries. Large viscosity ratios and large density ratios at the open boundary can cause severe stability difficulties [6].

While outflow/open boundary conditions for single-phase flows have been under intensive investigations for decades, very scarce work exists for two-phase outflows or open boundaries. In [7] the zero-flux (Neumann), convective, and extrapolation boundary conditions originated from single-phase flows have been studied for the two-phase lattice-Boltzmann equation. The zero-flux condition is also employed for the outflow boundary in [8] within the context of a coupled level-set/volume-of-fluids method, and in [9] in the context of a level set method where the outflow boundary involves only a single type of fluid. The outflow condition for two immiscible fluids in a porous medium is discussed in [10]. The works of [11, 12] have both considered the outflow condition for two-phase compressible flows in one dimension.

In a recent work [6] we have proposed a set of effective two-phase outflow (and also inflow) boundary conditions within the phase field framework. A salient characteristic of these boundary conditions is that they ensure the energy stability of the two-phase system. By looking into the two-phase energy balance relation, we have shown, at the continuum level, that with these boundary conditions the total energy of the two-phase system will not increase over time, even in situations where there are strong vortices or backflows, large viscosity contrast, and large density contrast at the outflow/open boundaries. In [6] we have further developed an algorithm for numerically treating these open boundary conditions based on a velocity-correction type splitting strategy.

In the context of *single-phase* incompressible Navier-Stokes equations, we have developed in another recent work [5] a general form of open boundary conditions that ensure the energy dissipation at the outflow/open boundary for *single-phase* flows. This general form represents a family of boundary conditions that are effective in dealing with the backflow instability at the single-phase open boundary, and its effectiveness has been demonstrated by extensive *single-phase* incompressible flow simulations in [5].

In the current paper we aim to extend the general single-phase open boundary conditions of [5] to *two-phase* outflows. Inspired by the general form of single-phase open boundary conditions from [5], we combine the ideas of [5] and [6] in this work and suggest several new forms of outflow/open boundary conditions for the *two-phase* momentum equations within the phase field framework. There exist some primary differences between two-phase and single-phase open boundary conditions, namely, the variable density/viscosity and the effect of surface tension in the two-phase case. Such complications are dealt with using a strategy similar to that of [6]. We also present an algorithm for numerically treating these new open boundary conditions. The current algorithm solves the two-phase momentum equations based on a rotational pressure-correction type splitting strategy. The main numerical challenge again lies in the variable density and variable viscosity of the two-phase mixture. By reformulating the pressure and viscous terms in the two-phase momentum equations, a strategy originally developed in [13], our algorithm gives rise to linear algebraic systems for the pressure and the velocity after discretization that involve only *constant* and *time-independent* coefficient matrices, despite the variable density and variable viscosity of the two-phase mixture. Therefore, these coefficient matrices can be pre-computed during pre-processing. This makes the current algorithm computationally very efficient and attractive. The current algorithm extends the pressure-correction strategy of [5] from single-phase to two-phase flows.

It is commonly observed that, with traditional splitting type schemes, the variable density in the Navier-Stokes equation has resulted in a variable (time-dependent) coefficient matrix for the pressure linear algebraic system after discretization [14–21]. This creates a severe computational and performance issue, due to the need for the frequent re-computation of the coefficient matrix and the challenge in efficiently solving the resultant linear algebraic system at large density ratios. Guermond & Salgado [22] have advocated a penalty point of view toward the projection idea, leading to a Poisson type equation for the pressure; see also [23, 24]. Dong & Shen [13] have proposed a different strategy for coping with the variable density. By a reformulation of the pressure term in the variable-density Navier-Stokes equation, they have developed a scheme which requires the solution of a pressure Poisson equation with constant (time-independent) coefficient matrix; see also [6, 25–28].

The algorithmic formulation presented in the current work is different from that of [6] for the two-phase momentum equations. The pressure correction strategy provides a different means to de-couple the computations for the velocity and pressure. With the rotational pressure correction strategy of the current work, after reformulating the pressure and viscous terms to treat the variable density and variable viscosity, we first compute an approximate velocity and an approximation of the divergence of this velocity, and then compute the pressure by projecting this approximate velocity to the space of divergence-free functions. In contrast, with the velocity-correction strategy of [6] one first computes the pressure by enforcing the divergence free condition on an approximation of the velocity, and then computes the velocity using the newly-computed pressure. In some sense, these different formulations for de-coupling the pressure and velocity can be considered complementary to each other. An exposition of the pressure-correction idea for the single-phase Stokes equations can be found in the review paper [29]. To implement the proposed open boundary conditions, our algorithm imposes a Neumann-type condition for the discrete velocity and a Dirichlet-type condition for the discrete pressure on the open/outflow boundary. Both of the discrete-velocity and discrete-pressure conditions stem from the open boundary conditions developed herein.

The contributions of this paper lie in two aspects: (i) the several new forms of outflow/open boundary conditions for the two-phase momentum equations, and (ii) the pressure-correction based algorithm for numerically treating these two-phase open boundary conditions. On the other hand, we would like to point out that the method for solving the phase field equation employed in the current paper is not new. It was originally developed in [13].

The numerical algorithm presented herein has been implemented using *C*^0^ continuous high-order spectral elements [30–32] for spatial discretizations in the current paper. It should however be noted that the algorithm is general and can also be implemented with other spatial discretization techniques.

## Pressure Correction Scheme for Two-Phase Outflows

In this section we present several new open boundary conditions for the two-phase momentum equations, together with an algorithm for numerically treating these open boundary conditions based on a rotational pressure correction-type strategy.

### Governing Equations and Open Boundary Conditions

Let Ω denote a flow domain in two or three dimensions (2-D or 3-D), and ∂Ω denote the boundary of Ω. Consider the mixture of two immiscible incompressible fluids contained in Ω. We use *ρ*_1_ and *ρ*_2_ to denote the constant densities of the two individual fluids, and use *μ*_1_ and *μ*_2_ to denote their constant dynamic viscosities. With the phase field approach, the motion of this mixture can be described by the following system of equations [13, 33],

$$\begin{array}{cc} & \rho \left(\frac{\partial u}{\partial t}+u\cdot \nabla u\right)=-\nabla p+\nabla \cdot \left[\mu D(u)\right]-\lambda \nabla \cdot \left(\nabla \phi ⊗\nabla \phi \right)+f \end{array}$$ (1a)

$$\begin{array}{c} \nabla \cdot u=0 \end{array}$$ (1b)

$$\begin{array}{c} \frac{\partial \phi }{\partial t}+u\cdot \nabla \phi =-\lambda \gamma_{1}\nabla^{2}\left[\nabla^{2}\phi -h\left(\phi \right)\right]+g\left(x,t\right) \end{array}$$ (1c)

where **x** and *t* are respectively the spatial coordinates and time, **u**(**x**, *t*) is the velocity, *p*(**x**, *t*) is pressure, **D**(**u**) = ∇**u** + ∇**u**^*T*^ (the superscript *T* denotes transpose), **f**(**x**, *t*) denotes some external body force, and ⊗ represents the tensor product. *ϕ*(**x**, *t*) is the phase field function, −1 ⩽ *ϕ* ⩽ 1. Regions with *ϕ* = 1 denote the first fluid, and the regions with *ϕ* = −1 denote the second fluid. The function *h*(*ϕ*) is given by $h\left(\phi \right)=\frac{1}{\eta^{2}}\phi \left(\phi^{2}-1\right),$ where *η* is the characteristic scale of the interfacial thickness. *λ* is referred to as the mixing energy density coefficient and is given by $\lambda =\frac{3}{2\sqrt{2}}\sigma \eta$[34], where *σ* is the surface tension and assumed to be constant in the current paper. The constant *γ*_1_ > 0 is the mobility coefficient associated with the interface. *ρ*(*ϕ*) and *μ*(*ϕ*) are respectively the density and dynamic viscosity of the mixture, given by

$$\begin{array}{c} \rho \left(\phi \right)=\frac{1}{2}\left(\rho_{1}+\rho_{2}\right)+\frac{1}{2}\left(\rho_{1}-\rho_{2}\right)\phi ,\;\mu \left(\phi \right)=\frac{1}{2}\left(\mu_{1}+\mu_{2}\right)+\frac{1}{2}\left(\mu_{1}-\mu_{2}\right)\phi . \end{array}$$ (2)

The function *g*(**x**, *t*) in Eq (1c) is a prescribed source term for the purpose of numerical testing only, and will be set to *g*(**x**, *t*) = 0 in actual simulations. Eq (1c) with *g* = 0 is the Cahn-Hilliard equation.

We assume that the domain boundary consists of three types which do not overlap with one another, ∂Ω = ∂Ω_*i*_ ∪ ∂Ω_*w*_ ∪ ∂Ω_*o*_. We refer to ∂Ω_*i*_ as the inflow boundary, ∂Ω_*w*_ as the wall boundary, and ∂Ω_*o*_ as the outflow or open boundary. On the inflow and the wall boundaries, the velocity **u** is assumed to be known. In addition, the phase field function *ϕ* is also assumed to be known on the inflow boundary. On the wall boundary we assume that the wettability property (i.e. contact angle) is known. On the other hand, at the outflow/open boundary ∂Ω_*o*_ none of the flow variables (velocity **u**, pressure *p*, phase field function *ϕ*) is known.

Inspired by the open boundary conditions for *single-phase incompressible flows* of [5] and the two-phase energy balance relation from [6], we suggest in the following several new forms of boundary conditions for the two-phase open boundary ∂Ω_*o*_:

$$\begin{array}{c} \begin{array}{cc} -pn+\mu n\cdot D(u) & -\left[\frac{\lambda }{2}\nabla \phi \cdot \nabla \phi +F\left(\phi \right)\right]n\\ & -\frac{1}{4}\rho \left[{\left|u\right|}^{2}n+\left(n\cdot u\right)u\right]\Theta_{0}\left(n,u\right)=f_{b}\left(x,t\right),\;\text{on}\;\partial \Omega_{o}; \end{array} \end{array}$$ (3a)

$$\begin{array}{c} \begin{array}{cc} -pn+\mu n\cdot D(u) & -\left[\frac{\lambda }{2}\nabla \phi \cdot \nabla \phi +F\left(\phi \right)\right]n\\ & -\frac{1}{2}\rho \left[{\left|u\right|}^{2}n+\left(n\cdot u\right)u\right]\Theta_{0}\left(n,u\right)=f_{b}\left(x,t\right),\;\text{on}\;\partial \Omega_{o}; \end{array} \end{array}$$ (3b)

$$\begin{array}{c} \begin{array}{cc} -pn+\mu n\cdot D(u) & -\left[\frac{\lambda }{2}\nabla \phi \cdot \nabla \phi +F\left(\phi \right)\right]n\\ & -\left[{\rho |u|}^{2}n\right]\Theta_{0}\left(n,u\right)=f_{b}\left(x,t\right),\;\text{on}\;\partial \Omega_{o}; \end{array} \end{array}$$ (3c)

$$\begin{array}{c} \begin{array}{cc} -pn+\mu n\cdot D(u) & -\left[\frac{\lambda }{2}\nabla \phi \cdot \nabla \phi +F\left(\phi \right)\right]n\\ & -\left[\rho (n\cdot u)u\right]\Theta_{0}\left(n,u\right)=f_{b}\left(x,t\right),\;\text{on}\;\partial \Omega_{o}. \end{array} \end{array}$$ (3d)

In the above Eqs (3a)–(3d), **n** is the outward-pointing unit vector normal to ∂Ω_*o*_, and |**u**| denotes the magnitude of **u**. *μ* and *ρ* are respectively the mixture dynamic viscosity and density given by Eq (2), and note that they are field variables and time-dependent. The function *F*(*ϕ*) is given by $F\left(\phi \right)=\frac{\lambda }{4\eta^{2}}{\left(1-\phi^{2}\right)}^{2},$ and note that $\left[\frac{\lambda }{2}\nabla \phi \cdot \nabla \phi +F\left(\phi \right)\right]$ is the free energy density of the two-phase system [33, 34]. **f**_*b*_ is a prescribed function on ∂Ω_*o*_ for the purpose of numerical testing only, and will be set to **f**_*b*_ = 0 in actual simulations. Θ_0_(**n**,**u**) is a smoothed step function whose form is given by $\Theta_{0}\left(n,u\right)=\frac{1}{2}(1-\tanh\frac{n\cdot u}{U_{0}\delta })$[4, 6], where *U*_0_ is the characteristic velocity scale, and *δ* > 0 is a constant that is sufficiently small. *δ* controls the sharpness of the smoothed step function, and Θ_0_ approaches the step function as *δ* → 0. When *δ* is sufficiently small, Θ_0_(**n**, **u**) essentially assumes the unit value where **n** ⋅ **u** < 0 and vanishes otherwise.

In contrast, the following open boundary condition was investigated in [6],

$$\begin{array}{c} -pn+\mu n\cdot D\left(u\right)-\left[\frac{\lambda }{2}\nabla \phi \cdot \nabla \phi +F\left(\phi \right)\right]n-\left[\frac{1}{2}\rho {\left|u\right|}^{2}n\right]\Theta_{0}\left(n,u\right)=0,\;\text{on}\;\partial \Omega_{o}. \end{array}$$ (4)

Another boundary condition,

$$\begin{array}{c} -pn+\mu n\cdot D\left(u\right)-\left[\frac{\lambda }{2}\nabla \phi \cdot \nabla \phi +F\left(\phi \right)\right]n-\left[\frac{1}{2}\rho \left(n\cdot u\right)u\right]\Theta_{0}\left(n,u\right)=0,\;\text{on}\;\partial \Omega_{o}, \end{array}$$ (5)

was also mentioned in [6].

The physical meanings of the boundary conditions Eqs (3a)–(3d) can be analogized to that of Eq (4) explained in [6]. Let us assume for now **f**_*b*_ = 0 and *δ* → 0 in the Θ_0_(**n**, **u**) function. In Eqs (3a)–(3d), the first two terms denote the fluid stress on the outflow/open boundary ∂Ω_*o*_, and the third term represents an effective stress exerting on ∂Ω_*o*_ induced by the free energy flux through ∂Ω_*o*_. On the other hand, the terms involving Θ_0_ can be considered as some effective stress induced by the kinetic energy *influx* into the domain through ∂Ω_*o*_, which take effect only in regions of backflow on ∂Ω_*o*_ (i.e. **n** ⋅ **u** < 0), due to the Θ_0_(**n** ⋅ **u**) function. The several open boundary conditions have imposed different forms for this effective stress. This effective stress is critical to overcoming the backflow instability at the two-phase outflow/open boundary; see [5] for the single-phase cases.

**Remarks:** We briefly mention the following more general boundary condition for ∂Ω_*o*_,

$$\begin{array}{c} -pn+\mu n\cdot D\left(u\right)-\left[\frac{\lambda }{2}\nabla \phi \cdot \nabla \phi +F\left(\phi \right)\right]n\\ -\frac{1}{2}\rho \left[\left(1-\theta +\beta_{1}\right)\left(n\cdot u\right)u+\left(\theta +\beta_{2}\right){\left|u\right|}^{2}n\right]\Theta_{0}\left(n,u\right)=0,\;\text{on}\;\partial \Omega_{o}, \end{array}$$ (6)

where 0 ⩽ *θ* ⩽ 1, *β*_1_ ⩾ 0, and *β*_2_ ⩾ 0 are constant parameters. This is the two-phase counterpart to the general form of single-phase open boundary conditions discussed in [5]. The boundary conditions Eqs (3a)–(3d), (4) and (5) are particular cases of Eq (6). For example, Eq (3a) corresponds to (*θ*, *β*_1_, *β*_2_) = (1/2, 0, 0) and Eq (3d) corresponds to (*θ*, *β*_1_, *β*_2_) = (0, 1, 0) in Eq (6).

The boundary conditions discussed so far on ∂Ω_*o*_ are for the momentum equations Eqs (1a) and (1b). In addition to them, one also needs to supply appropriate boundary conditions on ∂Ω_*o*_ for the phase field Eq (1c). Note that two independent conditions will be needed on each boundary, due to the fourth spatial order of Eq (1c). For the phase field function *ϕ*, on the outflow boundary ∂Ω_*o*_ we will employ the boundary conditions developed in [6]

$$\begin{array}{cc} & n\cdot \nabla \left[\nabla^{2}\phi -h\left(\phi \right)\right]=g_{a1}\left(x,t\right),\;\text{on}\;\partial \Omega_{o} \end{array}$$ (7a)

$$\begin{array}{cc} & n\cdot \nabla \phi =-D_{0}\frac{\partial \phi }{\partial t}+g_{a2}\left(x,t\right),\;\text{on}\;\partial \Omega_{o}, \end{array}$$ (7b)

where *g*_*a*1_ and *g*_*a*2_ are prescribed source terms on ∂Ω_*o*_ for the purpose of numerical testing only, and will be set to *g*_*a*1_ = 0 and *g*_*a*2_ = 0 in actual simulations. The constant *D*_0_ ⩾ 0 is a chosen non-negative constant, and $\frac{1}{D_{0}}$ plays the role of a convection velocity at the outflow boundary ∂Ω_*o*_.

The boundary conditions for the other types of boundaries (wall and inflow) will be set in accordance with previous works [6, 25]. We impose a Dirichlet condition for the velocity on the inflow and wall boundaries,

$$\begin{array}{c} u=w\left(x,t\right),\;\text{on}\;\partial \Omega_{i}\cup \partial \Omega_{w}, \end{array}$$ (8)

where **w** is the boundary velocity. For the phase field function, we impose the following condition from [6] on the inflow boundary,

$$\begin{array}{cc} & \phi =\phi_{b}\left(x,t\right),\;\text{on}\;\partial \Omega_{i}, \end{array}$$ (9a)

$$\begin{array}{cc} & \nabla^{2}\phi -h\left(\phi \right)=g_{b}\left(x,t\right),\;\text{on}\;\partial \Omega_{i}, \end{array}$$ (9b)

where *ϕ*_*b*_ denotes the distribution of the phase field function on the inflow boundary, and *g*_*b*_ is a prescribed source term for numerical testing only and will be set to *g*_*b*_ = 0 in actual simulations. On the wall boundary we employ the the following contact-angle condition [25], considering only the effect of the static contact angle,

$$\begin{array}{cc} & n\cdot \nabla \left[\nabla^{2}\phi -h\left(\phi \right)\right]=g_{c1}\left(x,t\right),\;\text{on}\;\partial \Omega_{w}, \end{array}$$ (10a)

$$\begin{array}{cc} & n\cdot \nabla \phi =\frac{3\sigma }{4\lambda }\cos\theta_{s}\left(1-\phi^{2}\right)+g_{c2}\left(x,t\right),\;\text{on}\;\partial \Omega_{w}, \end{array}$$ (10b)

where *θ*_*s*_ is the static (equilibrium) contact angle formed between the fluid interface and the wall measured on the side of the first fluid, *g*_*c*1_ and *g*_*c*2_ are two prescribed source terms for the purpose of the numerical testing only and will be set to *g*_*c*1_ = 0 and *g*_*c*2_ = 0 in actual simulations.

Finally, we assume that the following initial conditions for the velocity and the phase field function are known

$$\begin{array}{c} u\left(x,0\right)=u_{in}\left(x\right),\;\phi \left(x,0\right)=\phi_{in}\left(x\right), \end{array}$$ (11)

where the initial velocity **u**_*in*_ and the initial phase field function *ϕ*_*in*_ should be compatible with the above boundary conditions and the governing equations.

### Two-Phase Momentum Equations: Algorithm and Implementation

The system of Eqs (1a)–(1c), the boundary conditions Eqs (7a)–(10b), and one of the conditions among Eqs (3a)–(3d), together with the initial conditions Eq (11) for the velocity and the phase field function, constitute the overall system that need to be solved in numerical simulations. We next consider the numerical solution of this system.

Because the phase field Eq (1c) is coupled to the momentum Eqs (1a) and (1b) only through the convection term, it is possible and convenient to treat the momentum equations and the phase field equation individually. Indeed, by treating the convection term in Eq (1c) explicitly, one can de-couple the computation for the phase field function from those for the momentum equations. On can first solve Eq (1c) for the phase field function, and then solve the momentum equations for the pressure and the velocity.

In the following we will first concentrate on the momentum Eqs (1a) and (1b), together with the associated boundary conditions Eqs (3a)–(3d) for ∂Ω_*o*_ and Eq (8) for ∂Ω_*i*_ and ∂Ω_*w*_. We defer the discussion of the solution to the phase field equation to an Appendix. In subsequent discussions of this section we assume that the variables *ϕ* and ∇^2^ *ϕ* have been computed in appropriate ways and are already available.

To facilitate the following discussions we introduce an auxiliary pressure, $P=p+\frac{\lambda }{2}\nabla \phi \cdot \nabla \phi ,$ which will also be loosely called pressure where no confusion arises. Then Eq (1a) can be transformed into

$$\begin{array}{c} \frac{\partial u}{\partial t}+u\cdot \nabla u=-\frac{1}{\rho }\nabla P+\frac{1}{\rho }\nabla \mu \cdot D\left(u\right)+\frac{\mu }{\rho }\nabla^{2}u-\frac{\lambda }{\rho }\nabla^{2}\phi \nabla \phi +\frac{1}{\rho }f. \end{array}$$ (12)

We further re-write the open boundary conditions Eqs (3a)–(3d) into a unified compact form

$$\begin{array}{c} -Pn+\mu n\cdot D\left(u\right)-F\left(\phi \right)n-E\left(\rho ,n,u\right)=f_{b},\;\text{on}\;\partial \Omega_{o}, \end{array}$$ (13)

where

$$\begin{array}{c} E\left(\rho ,n,u\right)=\left\{\begin{array}{cc} \frac{1}{4}{\rho [|u|}^{2}n+\left(n\cdot u\right)u]\Theta_{0}\left(n,u\right), & \text{for}\;\text{boundary}\;\text{condition}\;(3\text{a});\\ \frac{1}{2}{\rho [|u|}^{2}n+\left(n\cdot u\right)u]\Theta_{0}\left(n,u\right), & \text{for}\;\text{boundary}\;\text{condition}\;(3\text{b});\\ {\rho |u|}^{2}n\Theta_{0}\left(n,u\right), & \text{for}\;\text{boundary}\;\text{condition}\;(3\text{c});\\ \rho \left(n\cdot u\right)u\Theta_{0}\left(n,u\right), & \text{for}\;\text{boundary}\;\text{condition}\;(3\text{d}); \end{array}\right. \end{array}$$ (14)

The following algorithm is for the Eqs (12) and (1b), together with the boundary conditions Eq (8) on ∂Ω_*i*_ ∪ ∂Ω_*w*_ and Eq (13) on ∂Ω_*o*_. Note that the variables *ϕ* and ∇^2^ *ϕ* are assumed to be known here, as discussed before.

Let *n* denote the time step index, and (⋅)^*n*^ denote the variable (⋅) at time step *n*. We use **ũ**^*n*^ and **u**^*n*^ to denote two slightly different approximations of the velocity at time step *n*. Define **ũ**^0^ and *ϕ*^0^ = *ϕ*_*in*_. By enforcing Eq (12) at time step zero, one can compute the initial pressure *P*^0^ as follows. Let

$$\begin{array}{c} H_{p0}^{1}\left(\Omega \right)=\left\{\;v\in H^{1}\left(\Omega \right)\;:\;v{|}_{\partial \Omega_{o}}=0\;\right\}, \end{array}$$ (15)

and $q\in H_{p0}^{1}\left(\Omega \right)$ denote the test function. By taking the inner product between ∇*q* and Eq (12) and integrating by part, one obtains the weak form about *P*^0^,

$$\begin{array}{c} \int_{\Omega }\frac{1}{\rho^{0}}\nabla P^{0}\cdot \nabla q\\ =\int_{\Omega }\left[\frac{1}{\rho^{0}}f^{0}-{\tilde{u}}^{0}\cdot \nabla {\tilde{u}}^{0}+\frac{1}{\rho^{0}}\nabla \mu^{0}\cdot D\left({\tilde{u}}^{0}\right)-\frac{\lambda }{\rho^{0}}\Psi \nabla \phi^{0}+\nabla \left(\frac{\mu^{0}}{\rho^{0}}\right)\times {\tilde{\omega }}^{0}\right]\cdot \nabla q\\ -\int_{\partial \Omega_{i}\cup \partial \Omega_{w}\cup \partial \Omega_{o}}\frac{\mu^{0}}{\rho^{0}}n\times {\tilde{\omega }}^{0}\cdot \nabla q-\int_{\partial \Omega_{i}\cup \partial \Omega_{w}}n\cdot {\left.\frac{\partial w}{\partial t}\right|}^{0}q,\;\forall q\in H_{p0}^{1}\left(\Omega \right), \end{array}$$ (16)

where

$$\begin{array}{c} \rho^{0}=\rho \left(\phi^{0}\right),\;\mu^{0}=\mu \left(\phi^{0}\right),\;{\tilde{\omega }}^{0}=\nabla \times {\tilde{u}}^{0}. \end{array}$$ (17)

${\frac{\partial w}{\partial t}|}^{0}$ is the time derivative at time step zero, which can be numerically computed based on the second-order backward differential formula (BDF2) because the boundary velocity **w**(**x**, *t*) is known on ∂Ω_*i*_ ∪ ∂Ω_*w*_. Ψ represents the projection of ∇^2^ *ϕ*^0^ into the *H*^1^(Ω) space, and is given by the following weak form (*φ* denoting the test function),

$$\begin{array}{c} \int_{\Omega }\Psi \varphi =-\int_{\Omega }\nabla \phi^{0}\cdot \nabla \varphi +\int_{\partial \Omega_{i}\cup \partial \Omega_{w}\cup \partial \Omega_{o}}\left(n\cdot \nabla \phi^{0}\right)\varphi ,\;\forall \varphi \in H^{1}\left(\Omega \right). \end{array}$$ (18)

The weak forms Eqs (16) and (18) can be discretized in space using *C*^0^ spectral elements (or finite elements). We solve Eq (16), together with the Dirichlet condition

$$\begin{array}{c} P^{0}=\mu^{0}n\cdot D\left({\tilde{u}}^{0}\right)\cdot n-F\left(\phi^{0}\right)-n\cdot E\left(\rho^{0},n,{\tilde{u}}^{0}\right)-f_{b}^{0}\cdot n,\;\text{on}\;\partial \Omega_{o}, \end{array}$$ (19)

to obtain the initial pressure *P*^0^, where Ψ is obtained by solving Eq (18).

Given $\left({\tilde{u}}^{n},u^{n},P^{n},\phi^{n+1},\nabla^{2}\phi^{n+1}\right)$, where *ϕ*^*n*+1^ and ∇^2^ *ϕ*^*n*+1^ are assumed known and result from the algorithm for the phase field equation to be discussed later, we compute **ũ**^*n*+1^, **u**^*n*+1^ and *P*^*n*+1^, together with an auxiliary field variable *ξ*^*n*+1^, successively in a de-coupled fashion as follows:

For **ũ**^*n*+1^:

$$\begin{array}{c} \begin{array}{cc} & \frac{\gamma_{0}{\tilde{u}}^{n+1}-\hat{u}}{\Delta t}+{\tilde{u}}^{*,n+1}\cdot \nabla {\tilde{u}}^{*,n+1}+\frac{1}{\rho_{m}}\nabla P^{n}-\nu_{m}\nabla^{2}{\tilde{u}}^{n+1}\\ & \;=\left(\frac{1}{\rho_{m}}-\frac{1}{\rho^{n+1}}\right)\nabla P^{*,n+1}+\frac{1}{\rho^{n+1}}\nabla \mu^{n+1}\cdot D\left({\tilde{u}}^{*,n+1}\right)\\ & \;-\left(\frac{\mu^{n+1}}{\rho^{n+1}}-\nu_{m}\right)\nabla \times \nabla \times {\tilde{u}}^{*,n+1}-\frac{\lambda }{\rho^{n+1}}\nabla^{2}\phi^{n+1}\nabla \phi^{n+1}+\frac{1}{\rho^{n+1}}f^{n+1} \end{array} \end{array}$$ (20a)

$$\begin{array}{cc} & {\tilde{u}}^{n+1}=w^{n+1},\;\text{on}\;\partial \Omega_{i}\cup \partial \Omega_{w} \end{array}$$ (20b)

$$\begin{array}{c} \begin{array}{cc} & n\cdot D\left({\tilde{u}}^{n+1}\right)=\left(1-\frac{\mu^{n+1}}{\mu_{0}}\right)n\cdot D\left({\tilde{u}}^{*,n+1}\right)\\ & \;+\frac{1}{\mu_{0}}\left[P^{*,n+1}n+F\left(\phi^{n+1}\right)n+E\left(\rho^{n+1},n,{\tilde{u}}^{*,n+1}\right)+f_{b}^{n+1}\right],\;\;\text{on}\;\partial \Omega_{o} \end{array} \end{array}$$ (20c)

$$\begin{array}{cc} & n\cdot \nabla {\tilde{u}}^{n+1}=n\cdot D\left({\tilde{u}}^{n+1}\right)-n{\cdot (\nabla {\tilde{u}}^{*,n+1})}^{T},\;\text{on}\;\partial \Omega_{o}. \end{array}$$ (20d)

For *ξ*^*n*+1^:

$$\begin{array}{cc} & \frac{\gamma_{0}}{\Delta t}\xi^{n+1}-\nu_{m}\nabla^{2}\xi^{n+1}=\nabla \cdot G^{n+1}+\nabla \left(\frac{\mu^{n+1}}{\rho^{n+1}}\right)\cdot \nabla \times \nabla \times {\tilde{u}}^{n+1} \end{array}$$ (21a)

$$\begin{array}{c} \begin{array}{cc} & n\cdot \nabla \xi^{n+1}=\frac{1}{\nu_{m}}n\cdot \frac{\gamma_{0}w^{n+1}-\hat{w}}{\Delta t}-\frac{1}{\nu_{m}}n\cdot G^{n+1}\\ & \;\;\;\;+\frac{1}{\nu_{m}}\frac{\mu^{n+1}}{\rho^{n+1}}n\cdot \nabla \times \nabla \times {\tilde{u}}^{n+1},\;\text{on}\;\partial \Omega_{i}\cup \partial \Omega_{w} \end{array} \end{array}$$ (21b)

$$\begin{array}{cc} & \xi^{n+1}=\nabla \cdot {\tilde{u}}^{n+1},\;\text{on}\;\partial \Omega_{o}. \end{array}$$ (21c)

For *P*^*n*+1^:

$$\begin{array}{cc} & \frac{\gamma_{0}u^{n+1}-\gamma_{0}{\tilde{u}}^{n+1}}{\Delta t}+\frac{1}{\rho_{m}}\nabla \left(P^{n+1}-P^{n}+\rho_{m}\nu_{m}\xi^{n+1}\right)=0 \end{array}$$ (22a)

$$\begin{array}{cc} & \nabla \cdot u^{n+1}=0 \end{array}$$ (22b)

$$\begin{array}{cc} & n\cdot u^{n+1}=n\cdot w^{n+1},\;\text{on}\;\partial \Omega_{i}\cup \partial \Omega_{w} \end{array}$$ (22c)

$$\begin{array}{c} \begin{array}{cc} & P^{n+1}=\mu^{n+1}n\cdot D\left({\tilde{u}}^{n+1}\right)\cdot n-F\left(\phi^{n+1}\right)\\ & \;\;\;\;-n\cdot E\left(\rho^{n+1},n,{\tilde{u}}^{n+1}\right)-\mu_{\min}\nabla \cdot {\tilde{u}}^{n+1},\;\text{on}\;\partial \Omega_{o}. \end{array} \end{array}$$ (22d)

The notation employed in the Eqs (20a)–(22d) is as follows. Let *J* (*J* = 1 or 2) denote the temporal order of the scheme, and *χ* denote a generic variable. Then in the above equations, χ*^, *n*+1^ is a *J*-th order explicit approximation of *χ*^*n*+1^, given by

$$\begin{array}{c} \chi^{*,n+1}=\left\{\begin{array}{cc} \chi^{n}, & J=1\\ 2\chi^{n}-\chi^{n-1}, & J=2. \end{array}\right. \end{array}$$ (23)

The expression $\frac{1}{\Delta t}\left(\gamma_{0}\chi^{n+1}-\hat{\chi }\right)$ denotes an approximation of ${\frac{\partial \chi }{\partial t}|}^{n+1}$ by the *J*-th order backward differentiation formula, where Δ*t* is the time step size and

$$\begin{array}{c} \hat{\chi }=\left\{\begin{array}{cc} \chi^{n}, & J=1\\ 2\chi^{n}-\frac{1}{2}\chi^{n-1}, & J=2, \end{array}\right.\;\gamma_{0}=\left\{\begin{array}{cc} 1, & J=1\\ \frac{3}{2}, & J=2. \end{array}\right. \end{array}$$ (24)

In Eqs (21a) and (21b) **G**^*n*+1^ is given by

$$\begin{array}{c} G^{n+1}=\frac{1}{\rho^{n+1}}f^{n+1}-{\tilde{u}}^{*,n+1}\cdot \nabla {\tilde{u}}^{*,n+1}-\frac{1}{\rho_{m}}\nabla P^{n}+\left(\frac{1}{\rho_{m}}-\frac{1}{\rho^{n+1}}\right)\nabla P^{*,n+1}\\ +\frac{1}{\rho^{n+1}}\nabla \mu^{n+1}\cdot D\left({\tilde{u}}^{*,n+1}\right)-\frac{\lambda }{\rho^{n+1}}\nabla^{2}\phi^{n+1}\nabla \phi^{n+1}. \end{array}$$ (25)

The function **E**(*ρ*,**n**, **u**) is defined by Eq (14). *ρ*^*n*+1^ and *μ*^*n*+1^ are given by Eq (2) and by using *ϕ*^*n*+1^. In Eq (22d) *μ*_min_ = min(*μ*_1_, *μ*_2_).

In the above equations, *ρ*_*m*_ is a chosen constant that must satisfy the condition 0 < *ρ*_*m*_ ⩽ min(*ρ*_1_, *ρ*_2_). This condition is critical to the stability of the scheme. The scheme is observed to be unstable if this condition is violated. We will employ *ρ*_*m*_ = min(*ρ*_1_, *ρ*_2_) for the numerical simulations in later sections. *ν*_*m*_ is a chosen constant that is sufficiently large, and a reasonable condition is $\nu_{m}⩾\frac{1}{2}(\frac{\mu_{1}}{\rho_{1}}+\frac{\mu_{2}}{\rho_{2}}).$ *μ*_0_ in Eq (20c) is a chosen constant that is sufficiently large. In the presence of open boundaries and when *μ*_1_ ≠ *μ*_2_, the scheme is observed to be unstable if *μ*_0_ ⩽ min(*μ*_1_, *μ*_2_). We will use *μ*_0_ ⩾ max(*μ*_1_, *μ*_2_) in the numerical simulations in later sections. It is observed that increasing *ν*_*m*_ tends to improve the stability. Increasing *μ*_0_ also tends to improve the stability in the presence of open boundaries. Note that the constant *μ*_0_ here should not be confused with the field variable *μ*^0^ = *μ*(*ϕ*^0^) in Eq (17), which represents the distribution of the dynamic viscosity at time step zero.

We would like to make several comments on the above scheme:

- The computations for the pressure *P*^*n*+1^ and the velocity ${\tilde{u}}^{n+1}$ are de-coupled in this algorithm, and the velocity **u**^*n*+1^ can be evaluated based on Eq (22a) once *P*^*n*+1^ is computed.
- When discretizing the momentum Eq (12) we have first reformulated the pressure term and the viscous term as follows,
  $$\begin{array}{c} \left\{\begin{array}{cc} & \frac{1}{\rho }\nabla P\approx \frac{1}{\rho_{m}}\nabla P+\left(\frac{1}{\rho }-\frac{1}{\rho_{m}}\right)\nabla P^{*}\\ & \frac{\mu }{\rho }\nabla^{2}u=\nu_{m}\nabla^{2}u+\left(\frac{\mu }{\rho }-\nu_{m}\right)\nabla^{2}u\approx \nu_{m}\nabla^{2}u-\left(\frac{\mu }{\rho }-\nu_{m}\right)\nabla \times \nabla \times u^{*} \end{array}\right. \end{array}$$ (26)
  where P* and u* are respectively explicit approximations of *P* and **u** of a consistent order, and the identity ∇^2^ **u** = ∇(∇ ⋅ **u**) − ∇ × ∇ × **u** together with Eq (1b) has been used. The terms $(\frac{1}{\rho_{m}}-\frac{1}{\rho^{n+1}})\nabla P^{*,n+1}$ and $(\frac{\mu^{n+1}}{\rho^{n+1}}-\nu_{m})\nabla \times \nabla \times {\tilde{u}}^{*,n+1}$ in Eq (20a) arise from the above reformulations.
- The auxiliary variable *ξ*^*n*+1^ is an approximation of the quantity $\nabla \cdot {\tilde{u}}^{n+1}$. One can arrive at Eq (21a) by taking the divergence of Eq (20a) and noting that $\nabla \cdot \hat{u}=0$ thanks to Eqs (22b) and (24), and by replacing $\nabla \times \nabla \times {\tilde{u}}^{*,n+1}$ with $\nabla \times \nabla \times {\tilde{u}}^{n+1}$ in the resultant equation. This equation about *ξ*^*n*+1^ exists only in the discrete sense.
- The overall construction of the scheme resembles a rotational pressure-correction type strategy. The scheme is obtained in two steps: (i) reformulate the pressure and viscous terms of the momentum equation in the way as given by Eq (26) to treat the variable density and variable viscosity; (ii) employ a rotational pressure-correction strategy similar to that of [5] for incompressible Navier-Stokes equations on the reformulated two-phase system to de-couple the velocity and pressure computations. One can note that the scheme here contains features that distinguish it from the usual pressure-correction formulations [29]. Most notably, it involves a discrete equation and associated boundary conditions, Eqs (21a)–(21c), about the auxiliary variable *ξ*^*n*+1^. In addition, the pressure *P*^*n*+1^ from the current scheme resides in the *H*^1^(Ω) space. In contrast, the pressure from the usual pressure-correction formulations resides in the *L*^2^(Ω) space (see [29]).
- The scheme leads to linear algebraic systems involving only *constant* and *time-independent* coefficient matrices for the pressure, velocity, and the variable *ξ*^*n*+1^ after discretization. This is thanks to the reformulations of the pressure term and the viscous term, and the introduction of the constants *ρ*_*m*_ and *ν*_*m*_ in the scheme. The treatment of the pressure term for coping with the variable density is proposed by [13]. The treatment of the viscous term for dealing with the variable viscosity can be traced to the early works in the 1970s (e.g. [35]); see also other works in e.g. [13, 36]. Because only constant and time-independent coefficient matrices are involved, which can be pre-computed during pre-processing, the current scheme is computationally very attractive and efficient.
- In the velocity substep we impose a velocity Neumann-type condition, Eqs (20c) and (20d), on the open boundary ∂Ω_*o*_. The discrete condition Eq (20c) originates from the open boundary condition Eq (13). But it contains constructions involving the constant *μ*_0_, which are critical to the stability if open boundaries are present. In the absence of the *μ*_0_ constructions, the computation is unstable when the viscosity ratio of the two fluids becomes large and when the fluid interface passes through the open boundaries. The idea of the *μ*_0_ construction for treating the variable viscosity at the open boundary is first proposed by [6]. However, there exists a crucial difference in terms of stability between the current scheme and that of [6]. The algorithm of [6] is based on a velocity-correction type strategy, and it is observed that a smaller *μ*_0_ constant tends to improve the stability of that scheme in the presence of open boundaries [6]. In contrast, the current scheme is based on a pressure-correction type strategy, and we observe that a larger *μ*_0_ constant tends to improve the stability of the scheme when open boundaries are present.
- In the pressure substep we impose a pressure Dirichlet condition, Eq (22d), on the open boundary ∂Ω_*o*_. This discrete condition results essentially from taking the inner product between **n** and the open boundary condition Eq (13). However, note that it contains an extra term $\mu_{\min}\nabla \cdot {\tilde{u}}^{n+1}$ in the construction.

We employ *C*^0^ continuous spectral elements [30–32] for spatial discretizations in the current paper. Let us next consider how to implement the algorithm, represented by Eqs (20a)–(22d), using *C*^0^ spectral elements. The formulations presented below with no change also applies to *C*^0^ finite elements.

The main issue with regard to the implementation arises from the terms such as $\nabla \times \nabla \times {\tilde{u}}^{*,n+1},$ $\nabla \times \nabla \times {\tilde{u}}^{n+1},$ and ∇ ⋅ **G**^*n*+1^ involved in the algorithm. These terms cannot be directly computed in the discrete space of *C*^0^ elements. Note that the term ∇^2^ *ϕ*^*n*+1^ itself may also cause difficulty to *C*^0^ elements. However, this term will be computed in a proper fashion using *C*^0^ elements later when discussing how to solve the phase field equation. So here we assume that ∇^2^ *ϕ*^*n*+1^ is already available in a suitable form.

We will derive weak forms of the algorithm for different flow variables. In the process the terms causing difficulty to *C*^0^ elements will be treated in an appropriate way.

Let $\tilde{\omega }=\nabla \times \tilde{u}$ denote the vorticity. Eq (20a) can be re-written as

$$\begin{array}{c} \frac{\gamma_{0}}{\nu_{m}\Delta t}{\tilde{u}}^{n+1}-\nabla^{2}{\tilde{u}}^{n+1}=\frac{1}{\nu_{m}}\left[G^{n+1}+\frac{\hat{u}}{\Delta t}\right]-\frac{1}{\nu_{m}}\left(\frac{\mu^{n+1}}{\rho^{n+1}}-\nu_{m}\right)\nabla \times {\tilde{\omega }}^{*,n+1}, \end{array}$$ (27)

where **G**^*n*+1^ is given by Eq (25). Let *H*_*u*0_(Ω) = {*v* ∈ *H*^1^(Ω): *v*|_∂Ω_*i*_∪∂Ω_*w*__ = 0}, and $\varphi \in H_{u0}^{1}\left(\Omega \right)$ denote the test function. Taking the *L*^2^ inner product between *φ* and Eq (27), and integrating by part, we get the weak form about ${\tilde{u}}^{n+1}$,

$$\begin{array}{c} \begin{array}{cc} & \frac{\gamma_{0}}{\nu_{m}\Delta t}\int_{\Omega }\varphi {\tilde{u}}^{n+1}+\int_{\Omega }\nabla \varphi \cdot \nabla {\tilde{u}}^{n+1}\\ & =\frac{1}{\nu_{m}}\int_{\Omega }\left[G^{n+1}+\frac{\hat{u}}{\Delta t}+\nabla \left(\frac{\mu^{n+1}}{\rho^{n+1}}\right)\times {\tilde{\omega }}^{*,n+1}\right]\varphi \\ & -\frac{1}{\nu_{m}}\int_{\Omega }\left(\frac{\mu^{n+1}}{\rho^{n+1}}-\nu_{m}\right){\tilde{\omega }}^{*,n+1}\times \nabla \varphi -\frac{1}{\nu_{m}}\int_{\partial \Omega_{o}}\left(\frac{\mu^{n+1}}{\rho^{n+1}}-\nu_{m}\right)n\times {\tilde{\omega }}^{*,n+1}\varphi \\ & +\int_{\partial \Omega_{o}}\left\{\frac{1}{\mu_{0}}\left[P^{*,n+1}n+F\left(\phi^{n+1}\right)n+E\left(\rho^{n+1},n,{\tilde{u}}^{*,n+1}\right)+f_{b}^{n+1}\right]\right.\\ & \;\;\left.+\left(1-\frac{\mu^{n+1}}{\mu_{0}}\right)n\cdot D\left({\tilde{u}}^{*,n+1}\right)-n{\cdot (\nabla {\tilde{u}}^{*,n+1})}^{T}\right\}\varphi ,\;\forall \varphi \in H_{u0}^{1}\left(\Omega \right). \end{array} \end{array}$$ (28)

When deriving the above weak form we have used the Eqs (20c) and (20d), and the identity (*K* denoting a scalar field function)

$$\begin{array}{c} \int_{\Omega }K\left(\nabla \times \tilde{\omega }\right)\varphi =\int_{\partial \Omega }K\left(n\times \tilde{\omega }\right)\varphi -\int_{\Omega }\left(\nabla K\times \tilde{\omega }\right)\varphi +\int_{\Omega }K\left(\tilde{\omega }\times \nabla \varphi \right). \end{array}$$

Let $\vartheta \in H_{p0}^{1}\left(\Omega \right)$ denote the test function, where $H_{p0}^{1}\left(\Omega \right)$ is defined in Eq (15). Taking the *L*^2^ inner product between *ϑ* and Eq (21a), and integrating by part, we have

$$\begin{array}{c} \begin{array}{cc} & \frac{\gamma_{0}}{\nu_{m}\Delta t}\int_{\Omega }\xi^{n+1}\vartheta +\int_{\Omega }\nabla \xi^{n+1}\cdot \nabla \vartheta \\ & =-\frac{1}{\nu_{m}}\int_{\Omega }\left[G^{n+1}+\nabla \left(\frac{\mu^{n+1}}{\rho^{n+1}}\right)\times {\tilde{\omega }}^{n+1}\right]\cdot \nabla \vartheta \\ & +\frac{1}{\nu_{m}}\int_{\partial \Omega_{i}\cup \partial \Omega_{w}}n\cdot \frac{\gamma_{0}w^{n+1}-\hat{w}}{\Delta t}\vartheta +\frac{1}{\nu_{m}}\int_{\partial \Omega_{i}\cup \partial \Omega_{w}}\frac{\mu^{n+1}}{\rho^{n+1}}n\cdot \nabla \times {\tilde{\omega }}^{n+1}\vartheta \\ & -\frac{1}{\nu_{m}}\int_{\partial \Omega }\nabla \left(\frac{\mu^{n+1}}{\rho^{n+1}}\right)\cdot n\times {\tilde{\omega }}^{n+1}\vartheta ,\;\forall \vartheta \in H_{p0}^{1}\left(\Omega \right), \end{array} \end{array}$$ (29)

where we have used the fact that $\vartheta \in H_{p0}^{1}\left(\Omega \right)$, Eq (21b), the divergence theorem, and the identity (*K* denoting a scalar field function)

$$\begin{array}{c} \nabla K\cdot \nabla \times \tilde{\omega }\vartheta =\nabla \cdot \left(\tilde{\omega }\times \nabla K\vartheta \right)+\nabla K\cdot \left(\tilde{\omega }\times \nabla \vartheta \right). \end{array}$$

We note the relation

$$\begin{array}{c} \begin{array}{cc} & \int_{\partial \Omega_{i}\cup \partial \Omega_{w}}\frac{\mu }{\rho }n\cdot \nabla \times \tilde{\omega }\vartheta =\int_{\partial \Omega }\frac{\mu }{\rho }n\cdot \nabla \times \tilde{\omega }\vartheta \\ & =\int_{\partial \Omega }\nabla \left(\frac{\mu }{\rho }\right)\cdot n\times \tilde{\omega }\vartheta +\int_{\partial \Omega }\frac{\mu }{\rho }n\cdot \tilde{\omega }\times \nabla \vartheta ,\;\forall \vartheta \in H_{p0}^{1}\left(\Omega \right), \end{array} \end{array}$$ (30)

where we have used the fact $\vartheta \in H_{p0}^{1}\left(\Omega \right)$, and have repeatedly used the divergence theorem. Then, Eq (29) can be transformed into the final weak form about *ξ*^*n*+1^,

$$\begin{array}{c} \begin{array}{cc} & \frac{\gamma_{0}}{\nu_{m}\Delta t}\int_{\Omega }\xi^{n+1}\vartheta +\int_{\Omega }\nabla \xi^{n+1}\cdot \nabla \vartheta =-\frac{1}{\nu_{m}}\int_{\Omega }\left[G^{n+1}+\nabla \left(\frac{\mu^{n+1}}{\rho^{n+1}}\right)\times {\tilde{\omega }}^{n+1}\right]\cdot \nabla \vartheta \\ & \;\;+\frac{1}{\nu_{m}}\int_{\partial \Omega_{i}\cup \partial \Omega_{w}}n\cdot \frac{\gamma_{0}w^{n+1}-\hat{w}}{\Delta t}\vartheta +\frac{1}{\nu_{m}}\int_{\partial \Omega_{i}\cup \partial \Omega_{w}\cup \partial \Omega_{o}}\frac{\mu^{n+1}}{\rho^{n+1}}n\times {\tilde{\omega }}^{n+1}\cdot \nabla \vartheta ,\\ & \;\;\;\forall \vartheta \in H_{p0}^{1}\left(\Omega \right). \end{array} \end{array}$$ (31)

Let $q\in H_{p0}^{1}\left(\Omega \right)$ denote the test function. Taking the *L*^2^ inner product between ∇*q* and Eq (22a) and integrating by part, we obtain the weak form about *P*^*n*+1^,

$$\begin{array}{c} \int_{\Omega }\nabla P^{n+1}\cdot \nabla q=\int_{\Omega }\left[\frac{\gamma_{0}\rho_{m}}{\Delta t}{\tilde{u}}^{n+1}+\nabla \left(P^{n}-\rho_{m}\nu_{m}\xi^{n+1}\right)\right]\cdot \nabla q\\ -\frac{\gamma_{0}\rho_{m}}{\Delta t}\int_{\partial \Omega_{i}\cup \partial \Omega_{w}}n\cdot w^{n+1}q,\;\forall q\in H_{p0}^{1}\left(\Omega \right), \end{array}$$ (32)

where we have used the divergence theorem, and the Eqs (22b) and (22c).

One can observe that the weak forms Eqs (28), (31) and (32) involve no derivatives of order two or higher, and all the terms can be computed directly with *C*^0^ elements. These weak forms can be discretized in space using *C*^0^ spectral elements in the standard way [31].

Given $\left({\tilde{u}}^{n},u^{n},P^{n},\phi^{n+1},\nabla^{2}\phi^{n+1}\right)$, our final algorithm for solving the momentum equations therefore consists of the following procedure. We refer to this procedure as **AdvanceMomentum** hereafter. It produces $\left({\tilde{u}}^{n+1},u^{n+1},P^{n+1}\right)$ as follows:

**AdvanceMomentum procedure:**
- Solve Eq (28), together with the velocity Dirichlet condition Eq (20b) on ∂Ω_*i*_ ∪ ∂Ω_*w*_, for ${\tilde{u}}^{n+1}$;
- Solve Eq (31), together with the Dirichlet condition Eq (21c) on ∂Ω_*o*_, for *ξ*^*n*+1^;
- Solve Eq (32), together with the pressure Dirichlet condition Eq (22d) on ∂Ω_*o*_, for *P*^*n*+1^;
- Evaluate **u**^*n*+1^ based on Eq (22a) in the following form:
  $$\begin{array}{c} u^{n+1}={\tilde{u}}^{n+1}-\frac{\Delta t}{\gamma_{0}\rho_{m}}\nabla \left(P^{n+1}-P^{n}+\rho_{m}\nu_{m}\xi^{n+1}\right). \end{array}$$

In the above algorithm, when imposing the Dirichlet condition Eq (21c) about *ξ*^*n*+1^ on ∂Ω_*o*_ and when imposing the pressure Dirichlet condition Eq (22d) on ∂Ω_*o*_, it should be noted that with *C*^0^ elements one needs to first project the Dirichlet data computed from these equations into the *H*^1^(∂Ω_*o*_), and then impose the projected data as the Dirichlet condition. This is because the expressions for the boundary conditions of Eqs (21c) and (22d) involve derivatives, which may not be continuous across element boundaries on ∂Ω_*o*_ for *C*^0^ elements.

One can observe that the **AdvanceMomentum** algorithm has the following characteristics: (i) The computations for the velocity, the pressure, and the field variable *ξ*^*n*+1^ are all de-coupled; (ii) The computations for the different components of the velocity ${\tilde{u}}^{n+1}$ are de-coupled in Eq (28); (iii) All resultant linear algebraic systems from the algorithm involve only constant and time-independent coefficient matrices, which can be pre-computed.

As discussed in [13], the density *ρ*^*n*+1^ and the dynamic viscosity *μ*^*n*+1^ computed according to Eq (2) based on *ϕ*^*n*+1^ may encounter numerical difficulties when the density ratio between the two fluids becomes very large or conversely very small. This is because the numerically-computed *ϕ* may not exactly lie within the range [−1, 1] and may be slightly out of bound at certain spatial points in the domain, because of the interaction between mass conservation and the minimization of the free energy inherent in the Cahn-Hilliard dynamics. At large density ratios, the slightly out-of-range values of *ϕ* may cause the density or the dynamic viscosity computed from Eq (2) to become negative at certain points, thus causing numerical difficulties. Following [13], when the density ratio becomes large or conversely small (typically beyond 10^2^ or below 10^−2^), we will use the following modified function for computing the mixture density and dynamic viscosity,

$$\begin{array}{c} \tilde{\phi }=\left\{\begin{array}{cc} \phi , & \text{if}\;|\phi |⩽1,\\ \text{sign}(\phi ), & \text{if}\;|\phi |>1; \end{array}\right.\;\left\{\begin{array}{cc} & \rho =\frac{1}{2}\left(\rho_{1}+\rho_{2}\right)+\frac{1}{2}\left(\rho_{1}-\rho_{2}\right)\tilde{\phi },\\ & \mu =\frac{1}{2}\left(\mu_{1}+\mu_{2}\right)+\frac{1}{2}\left(\mu_{1}-\mu_{2}\right)\tilde{\phi }. \end{array}\right. \end{array}$$ (33)

### Overall Method for Two-Phase Flow Simulations

Let us now consider the numerical solution of the phase field Eq (1c), together with the boundary conditions Eqs (9a) and (9b) for ∂Ω_*i*_, Eqs (10a) and (10b) for ∂Ω_*w*_, and Eqs (7a) and (7b) for ∂Ω_*o*_.

The fourth spatial order of the phase field Eq (1c) presents a special challenge to *C*^0^ spectral element type spatial discretizations (which we employ in the current work) and the usual finite element type methods, because derivatives of order two or higher cannot be directly computed in the discrete function space of *C*^0^ spectral and finite elements. This is unlike some other discretizations such as finite difference or spectral methods (see e.g. [33, 36]). In a previous work [13], we have developed an algorithm for the phase field Eq (1c). This algorithm computes the phase field function *ϕ*^*n*+1^ and ∇^2^ *ϕ*^*n*+1^ (both in *H*^1^(Ω) space) by solving two Helmholtz type equations in a successive but un-coupled fashion. It is particularly suitable for *C*^0^ spectral element (and also usual finite element) type spatial discretizations, and it has a low computational cost because the two Helmholtz equations are de-coupled. In contrast, with mixed formulations one will need to solve a system of two coupled 2nd-order equations (see e.g. [23, 37]), leading to increased computational costs.

We will employ the algorithm of [13] for the phase field equation in the current work. For the sake of completeness, we provide a summary of this algorithm for solving the phase field equation together with the boundary conditions in the Appendix of this paper, and it is referred to as the **AdvancePhase** procedure (see the Appendix).

Our overall method for simulating incompressible two-phase flows is a combination of the algorithm presented in the previous subsection for the momentum equations and the algorithm in the Appendix for the phase field equation. Specifically, given $\left({\tilde{u}}^{n},u^{n},P^{n},\phi^{n}\right)$, the overall discrete formulation of the method consists of Eqs (36a)–(36h) (in the Appendix), Eqs (20a)–(20d), (21a)–(21c) and (22a)–(22d). With *C*^0^ spectral-element spatial discretizations, we go through the developments discussed in the previous subsection and in the Appendix to obtain the weak forms for the field variables. The final solution procedure is composed of the following steps:

- Compute *ϕ*^*n*+1^ and ∇^2^ *ϕ*^*n*+1^ based on the **AdvancePhase** procedure discussed in the Appendix.
- Compute *ρ*^*n*+1^ and *μ*^*n*+1^ according to Eq (2) by using *ϕ*^*n*+1^ computed above. When the density ratio becomes large or conversely small (typically above 10^2^ or below 10^−2^), use Eq (33) instead.
- Compute $\left({\tilde{u}}^{n+1},u^{n+1},P^{n+1}\right)$ based on the **AdvanceMomentum** procedure discussed in the previous subsection, using *ϕ*^*n*+1^, ∇^2^ *ϕ*^*n*+1^, *ρ*^*n*+1^, and *μ*^*n*+1^ computed above.

It can be observed that this method has the following characteristics: (1) The computations for all the flow variables and auxiliary variables are completely de-coupled; (2) All the resultant linear algebraic systems after discretization involve only *constant* and *time-independent* coefficient matrices, which can be pre-computed; (3) Within each time step, the method involves only the solution of individual Helmholtz-type (including Poisson) equations. We observe that the method is effective for problems with large density ratios and large viscosity ratios at the two-phase outflow/open boundary.

## Representative Numerical Tests

In this section we demonstrate the accuracy of our method and its capability for coping with two-phase open boundaries. The test problems are in two dimensions, and they involve two-phase open boundaries, and large contrasts in densities and dynamic viscosities of the two fluids. We compare simulation results with the experimental measurement and with the exact physical solutions from theory to demonstrate that our method produces physically accurate results.

We first briefly mention the normalization of the governing equations and physical parameters, which has been discussed at length in previous works [6, 25]. Let *L* denote the characteristic length scale and *U*_0_ denote the characteristic velocity scale. In Table 1 we list the normalization constants for different physical variables and parameters. For instance, the non-dimensional mixing energy density coefficient is given by $\frac{\lambda }{\rho_{1}U_{0}^{2}L^{2}}$ based on this table. When the flow variables and parameters are normalized as given by the table, the forms of the governing equations and the boundary conditions will remain unchanged upon normalization. In the following discussions all the flow variables and physical parameters are given in non-dimensional forms unless otherwise noted, with the understanding that they have all been properly normalized.

**Table 1:** Normalization constants for the flow variables and parameters.

variables	normalization constants	variables	normalization constants
**x**, *η*	*L*	*σ*	$\rho_{1}U_{0}^{2}L$
**u**, **u**_*in*_, **w**	*U*_0_	*D*_0_	1/*U*_0_
*t*, Δ*t*	*L*/*U*_0_	*γ*_1_	*L*/(*ρ*_1_ *U*_0_)
**g**_*r*_ (gravity)	$U_{0}^{2}/L$	*λ*	$\rho_{1}U_{0}^{2}L^{2}$
*p*, *P*, **f**_*b*_	$\rho_{1}U_{0}^{2}$	*ν*_*m*_	*U*_0_ *L*
*ϕ*, $\hat{\phi }$, *ϕ*_*b*_, *ϕ*_*in*_, *θ*_*s*_	1	**f**	$\rho_{1}U_{0}^{2}/L$
*ρ*, *ρ*_1_, *ρ*_2_, *ρ*_*m*_	*ρ*_1_	*ξ*^*n*^, *g*	*U*_0_/*L*
*μ*, *μ*_1_, *μ*_2_, *μ*_0_	*ρ*_1_ *U*_0_ *L*	*g*_*a*1_	1/*L*^4^
*g*_*a*2_, *g*_*c*2_	1/*L*	*g*_*b*_	1/*L*^2^
*g*_*c*1_	1/*L*^3^		

### Convergence Rates

The goal here is to study the convergence behavior of the method developed herein and to demonstrate its spatial and temporal convergence rates using a contrived analytic solution.

**Fig 1:**
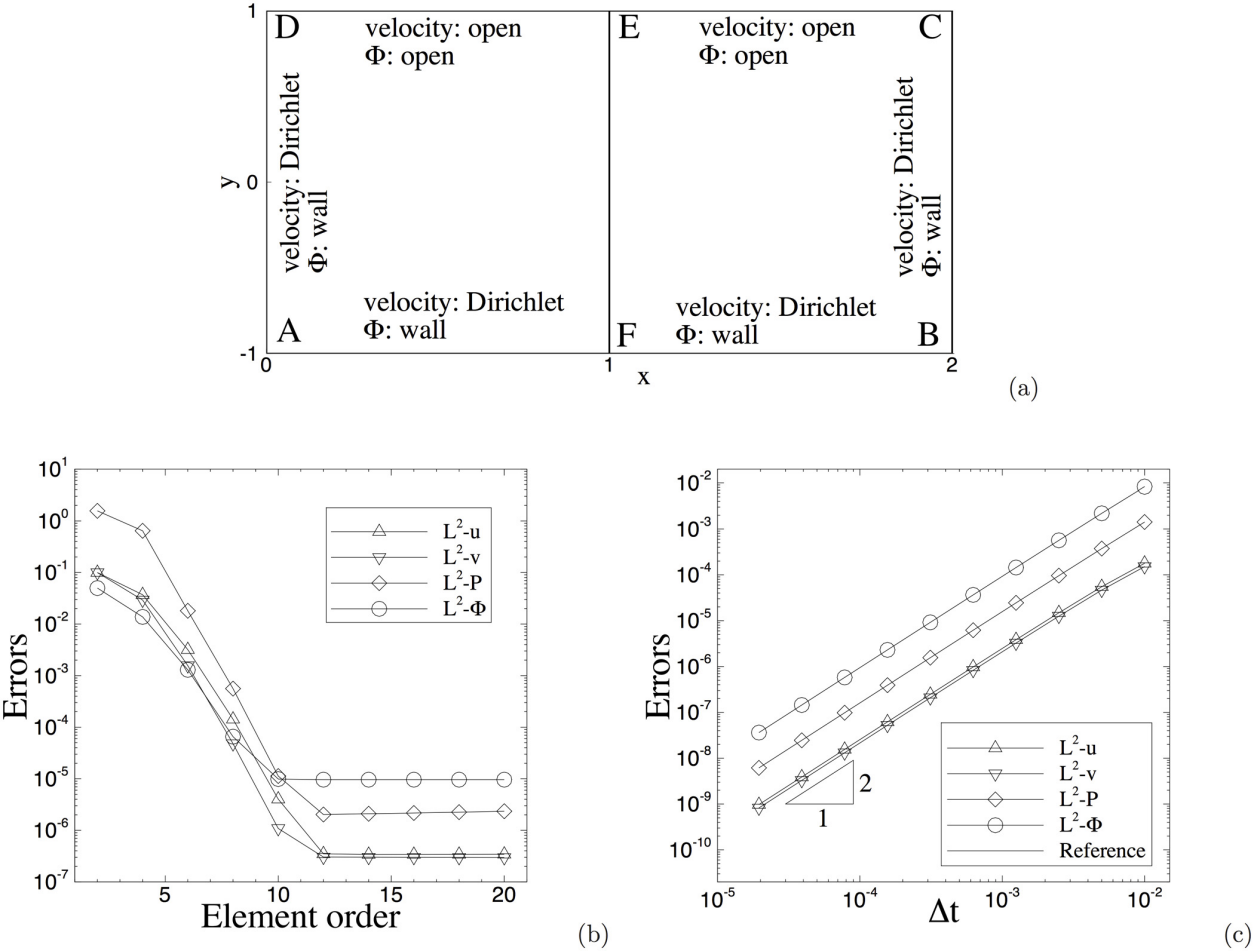
Spatial/temporal convergence rates: (a) Mesh and boundary conditions; (b) Numerical errors versus element order showing spatial exponential convergence (with fixed Δ*t* = 0.001); (c) Numerical errors versus Δ*t* showing temporal second-order convergence rate (element order fixed at 18). On the face $\bar{CD}$ the open boundary condition Eq (3b) is used.

Here is the problem setup. Fig 1(a) shows the rectangular domain $\bar{ABCD}$ for this problem, 0 ⩽ *x* ⩽ 2 and −1 ⩽ *y* ⩽ 1. We consider the following analytic expressions for the flow variables

$$\begin{array}{c} \left\{\begin{array}{cc} & u=A\cos\pi y\sin ax\sin Wt,\\ & v=-\frac{Aa}{\pi }\sin\pi y\cos ax\sin Wt, \end{array}\right.\;\left\{\begin{array}{cc} & P=A\sin\pi y\sin ax\cos Wt,\\ & \phi =B\cos a_{1}x\cos b_{1}y\sin W_{1}t, \end{array}\right. \end{array}$$ (34)

where (*u*, *v*) are the *x* and *y* velocity components, and *A*, *B*, *a*, *W*, *a*_1_, *b*_1_ and *W*_1_ are prescribed constants to be specified below. The *u* and *v* expressions evidently satisfy the Eq (1b). The external force **f**(**x**, *t*) in Eq (12) and the source term *g*(**x**, *t*) in Eq (1c) are chosen such that the expressions in Eq (34) satisfy the Eqs (12) and (1c).

For the boundary conditions, on the sides $\bar{AD}$, $\bar{AB}$ and $\bar{BC}$ we impose the velocity Dirichlet condition Eq (8) with the boundary velocity **w** chosen according to the analytic expressions of Eq (34), and we impose the contact-angle conditions Eqs (10a) and (10b) for the phase field function, in which *θ*_*s*_ = 90° and *g*_*c*1_ and *g*_*c*2_ are chosen such that the *ϕ* expression in Eq (34) satisfies Eqs (10a) and (10b). On the side $\bar{CD}$ we impose the open boundary condition Eq (13), in which **f**_*b*_ is chosen such that the analytic expressions in Eq (34) satisfy Eq (13), and we impose the conditions Eqs (7a) and (7b) for the phase field function, in which *D*_0_ = 0 and *g*_*a*1_ and *g*_*a*2_ are chosen such that the *ϕ* expression in Eq (34) satisfies Eqs (7a) and (7b). For the initial conditions Eq (11) we choose **u**_*in*_ and *ϕ*_*in*_ according to the analytic expressions in Eq (34) by setting *t* = 0.

We partition the domain along the *x* direction using two quadrilateral spectral elements of the same size as shown in Fig 1(a). The system of governing Eqs (12), (1b) and (1c) are integrated over time with the algorithm developed herein from *t* = 0 to *t* = *t*_*f*_ (*t*_*f*_ to be specified below). Then we compute and monitor the errors of the simulation results at *t* = *t*_*f*_ against the analytic solution given in Eq (34). The parameters for this problem are listed in Table 2.

**Table 2:** Parameter values for convergence tests.

parameters	values	parameters	values
*A*	2.0	*ρ*_*m*_	min(*ρ*_1_, *ρ*_2_)
*B*	1.0	*ν*_*m*_	$\frac{1}{2}(\frac{\mu_{1}}{\rho_{1}}+\frac{\mu_{2}}{\rho_{2}})$
*a*, *a*_1_, *b*_1_	*π*	*μ*_0_	max(*μ*_1_, *μ*_2_)
*W*, *W*_1_	1.0	*δ*	$\frac{1}{20}$
*ρ*_1_	1.0	*η*	0.1
*ρ*_2_	3.0	*θ*_*s*_	90°
*μ*_1_	0.01	*D*_0_	0.0
*μ*_2_	0.05	*J* (integration order)	2
*σ*	9.428 × 10^−2^		
*γ*_1_	0.01		

In the first group of tests we fix the final time at *t*_*f*_ = 0.1 and the time step size at Δ*t* = 0.001. Then we vary the element order systematically between 2 and 20. Fig 1(b) shows the *L*^2^ errors of the flow variables at *t* = *t*_*f*_ as a function of the element order. The results correspond to the open boundary condition Eq (3b). The numerical errors decrease exponentially as the element order increases (when below order 10). As the element order increases beyond 12, the error curves level off due to the saturation by the temporal truncation error.

In the second group of tests we fix the final integration time at *t*_*f*_ = 0.1 and the element order at a large value 18, and then vary the time step size systematically between Δ*t* = 1.953125 × 10^−5^ and Δ*t* = 0.01. In Fig 1(c) we plot the *L*^2^ errors of the flow variables as a function of Δ*t* in logarithmic scales. A slope of 2 has been observed in the error curves when the time step size becomes small.

The results of these tests demonstrate that the method developed in this work has a spatial exponential convergence rate and a temporal second-order convergence rate.

### Capillary Wave

The goal of this section is to demonstrate the physical accuracy of our method using a two-phase capillary wave problem, whose exact physical solution is known [38]. This problem involves two fluid phases, density contrast, viscosity contrast, gravity and the surface tension effects. We have considered this problem in a previous work [13]. It should be noted that the algorithm tested here is different from that of [13].

Here is the problem setting. We consider two immiscible incompressible fluids in an infinite domain. The lighter fluid occupies the top half of the domain, and the heavier fluid occupies the bottom half. The gravity is in the vertical direction and points downward. Without loss of generality we assume that the first fluid is lighter than the second one (*ρ*_1_ ⩽ *ρ*_2_). At *t* = 0, the interface formed between them is perturbed by a small-amplitude sinusoidal wave from its equilibrium horizontal position, and starts to oscillate. The goal is to study the behavior of the interface over time.

Prosperetti [38] reported an exact standing-wave (but time-dependent) solution to this problem under the following condition: The two fluids may have different densities and dynamic viscosities, but their kinematic viscosities must match. The capillary-wave amplitude versus time has been provided in [38]. We will simulate this problem under the same condition, and compare with the exact solution from [38].

**Fig 2:**
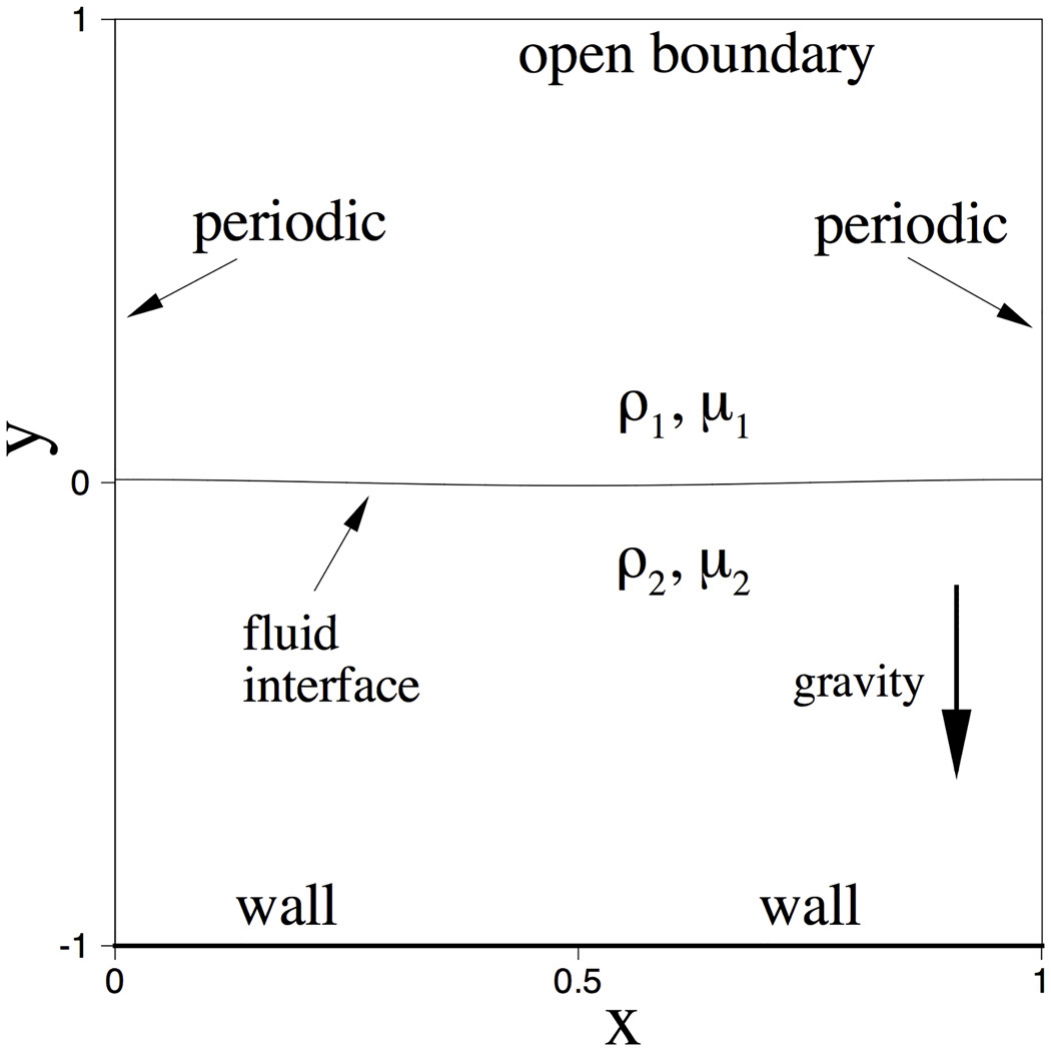
Configuration for the capillary wave problem.

Specifically, we consider a computational domain as depicted in Fig 2 (non-dimensionalized), 0 ⩽ *x* ⩽ 1 and −1 ⩽ *y* ⩽ 1. The un-perturbed equilibrium position of the fluid interface coincides with the *x*-axis. We assume that the initial perturbation profile of the interface is given by *y* = *H*_0_ cos *kx*, where $k=\frac{2\pi }{\lambda_{w}}=2\pi$ and *λ*_*w*_ = 1 is the wave length of the perturbation profile, and *H*_0_ = 0.01 is the initial amplitude. Note that the capillary wave-length *λ*_*w*_ is chosen to be the same as the domain dimension in the *x* direction, and that the initial capillary amplitude *H*_0_ is small compared to the domain dimension in the *y* direction.

We employ the algorithm developed here to solve the governing Eqs (12) and (1b)–(1c), where the external body force in Eq (12) is set to **f** = *ρ* **g**_*r*_ and **g**_*r*_ is the gravitational acceleration. For the boundary conditions, in the horizontal direction we assume that it is periodic at *x* = 0 and *x* = 1. At the bottom of the domain (*y* = −1), we assume a solid wall in the simulations, and impose the velocity Dirichlet condition Eq (8) with **w** = 0, and impose the boundary conditions Eqs (10a) and (10b) with *g*_*c*1_ = *g*_*c*2_ = 0 and *θ*_*s*_ = 90°. On the top side (*y* = 1) we assume that the domain is open, and impose the open boundary condition Eq (13) with **f**_*b*_ = 0 for the momentum equation, and impose the open boundary conditions Eqs (7a) and (7b) with *g*_*a*1_ = *g*_*a*2_ = 0 and *D*_0_ = 0 for the phase field function. We employ the following initial velocity and phase field function, **u**_*in*_(**x**) = 0 and $\phi_{in}\left(x\right)=\tanh(\frac{y-H_{0}\cos kx}{\sqrt{2}\eta }).$

We discretize the domain using 240 quadrilateral elements, with 10 elements in the *x* direction and 24 elements in the *y* direction. The elements are uniform along the *x* direction, and are non-uniform along the *y* direction, clustering around the region −0.012 ⩽ *y* ⩽ 0.012. We have used an element order 14 for all the elements.

We choose the physical parameters for this problem in accordance with those in [13]. A summary of the physical/numerical parameter values is provided in Table 3. Note that while *ρ*_2_ and *μ*_2_ are varied in different cases, the relation $\frac{\mu_{2}}{\rho_{2}}=\frac{\mu_{1}}{\rho_{1}}$ is maintained according to the condition of the exact solution by [38].

**Table 3:** Parameter values for the capillary wave problem.

parameters	values	parameters	values
|**g**_*r*_|	1.0	*ρ*_*m*_	min(*ρ*_1_, *ρ*_2_)
*σ*	1.0	*ν*_*m*_	$\frac{1}{2}(\frac{\mu_{1}}{\rho_{1}}+\frac{\mu_{2}}{\rho_{2}})$
*H*_0_	0.01	*μ*_0_	*μ*_1_
*λ*_*w*_	1.0	*δ*	1/100
*ρ*_1_	1.0	*D*_0_	0.0
*μ*_1_	0.01	*θ*_*s*_	90°
$\frac{\mu_{2}}{\rho_{2}}$	$\frac{\mu_{1}}{\rho_{1}}$	*J* (integration order)	2
*ρ*_2_, *μ*_2_	(varied)	Δ*t*	2.5 × 10^−5^
*η*	0.002	*λ*	$\frac{3}{2\sqrt{2}}\sigma \eta$
*γ*_1_	2.5*η*^2^	Element order	14
Number of elements in mesh	240		

**Fig 3:**
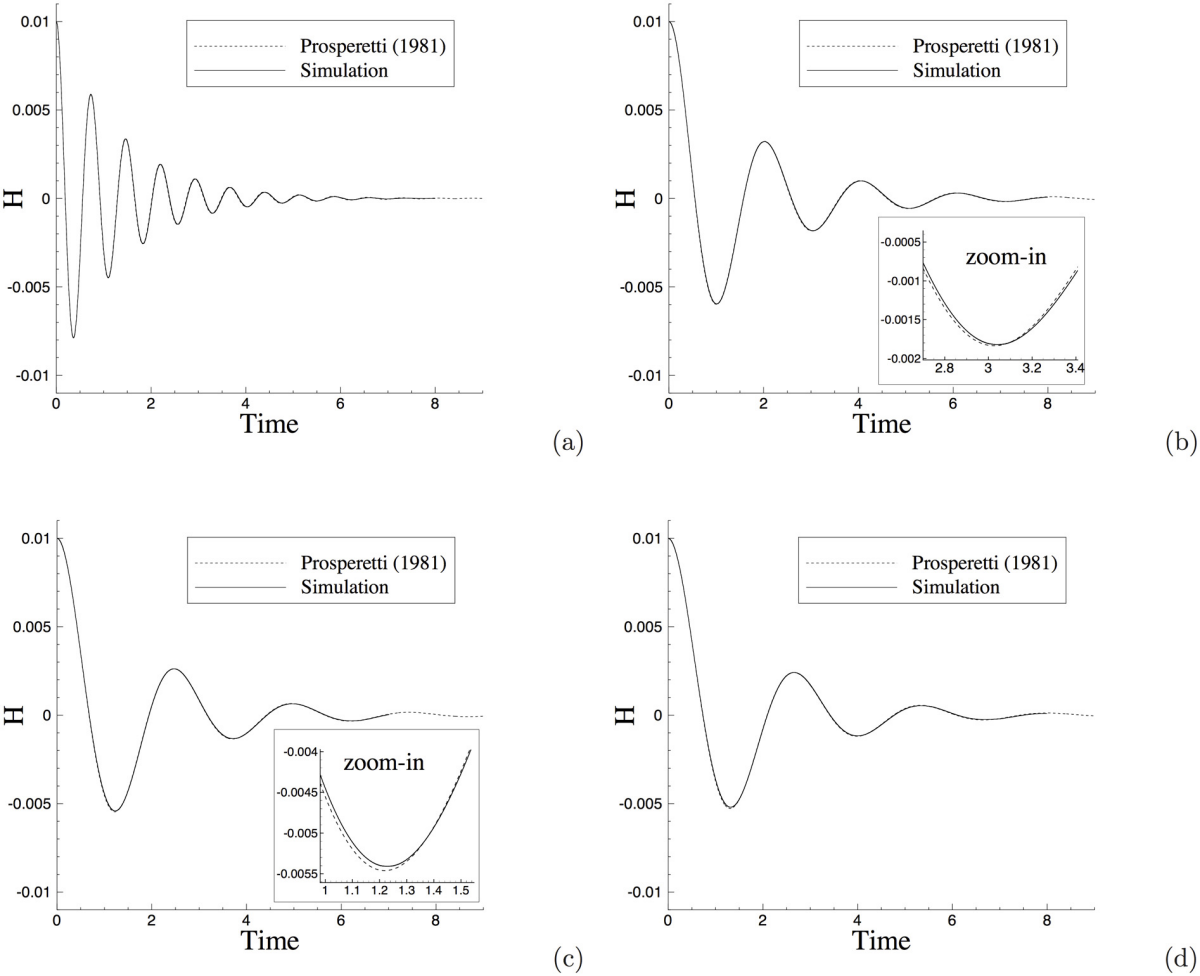
Comparison of time histories of the capillary-wave amplitudes between current simulation and the exact solution by Prosperetti [38] for density ratios (a) $\frac{\rho_{2}}{\rho_{1}}=2$, (b) $\frac{\rho_{2}}{\rho_{1}}=50$, (c) $\frac{\rho_{2}}{\rho_{1}}=200$, and (d) $\frac{\rho_{2}}{\rho_{1}}=1000$.

Let us compare the simulation results with the exact physical solution given by [38]. Fig 3 shows the time histories of the capillary amplitude *H*(*t*) from the simulation and from the exact solution [38] at several density ratios. Fig 3(a)–3(d) respectively corresponds to the density ratios $\frac{\rho_{2}}{\rho_{1}}=2$, 50, 200, and 1000. These results are obtained using the open boundary condition Eq (3b) at the upper domain boundary. It can be observed that the fluid interface fluctuates about its equilibrium position with the amplitude attenuated over time. The oscillation frequency decreases with increasing density ratios between the two fluids. One can further observe that the time-history curves from the simulations almost exactly overlap with those from the physical solution given by [38] for all density ratios. The insets of Fig 3(b) and 3(c) are the blow-up views of the curves, which show that the difference between the simulation and the exact physical solution is small. These results indicate that our method has produced physically accurate results for the capillary wave problem.

### Bouncing Water Drop on Superhydrophobic Surface

The goal of this section is to further evaluate the accuracy of the method developed here by comparing simulation results with the experimental measurement. The test problem considered in this section involves large density ratio, large viscosity ratio, and superhydrophobic walls (i.e. contact angle ⩾150°). A similar problem but under a different condition has been considered in a previous work [25].

**Fig 4:**
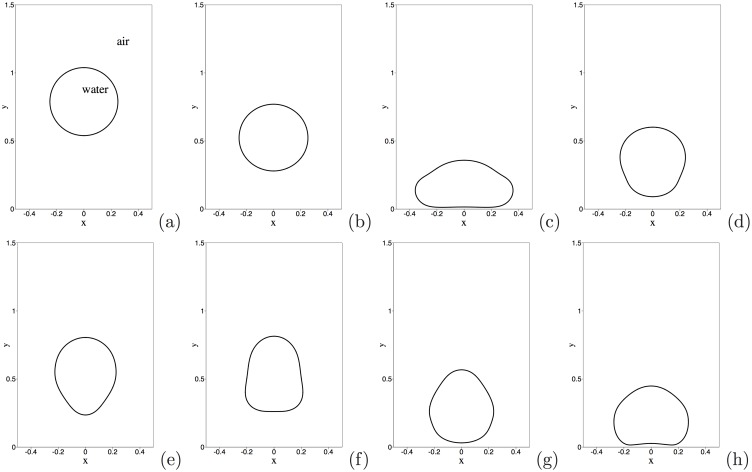
Bouncing water drop (drop radius 1mm, initial height *H*_0_ = 3.2mm): temporal sequence of snapshots of the air-water interface at non-dimensional time instants: (a) *t* = 0.05, (b) *t* = 0.25, (c) *t* = 0.4, (d) *t* = 0.55, (e) *t* = 0.7, (f) *t* = 0.85, (g) *t* = 1.0, (h) *t* = 1.15.

We consider a rectangular domain (see Fig 4(a)), $-\frac{L}{2}⩽x⩽\frac{L}{2}$ and $0⩽y⩽\frac{3L}{2}$, where *L* is specified later. The domain is periodic in the horizontal direction at $x=\pm \frac{L}{2}$. The top and bottom of the domain are two superhydrophobic solid walls. If the air-water interface intersects the walls, the contact angle is assumed to be 170°. The domain is initially filled with air. A water drop, initially circular with a radius $R_{0}=\frac{L}{4}$, is suspended in the air. The center of the water drop is initially located at a height *H*_0_ above the bottom wall, that is, (*x*_0_, *y*_0_) = (0, *H*_0_), where (*x*_0_, *y*_0_) is the coordinate of the center of mass of the water drop. *H*_0_ is varied in the simulations. The gravity is assumed to be in the −*y* direction. At *t* = 0, the water drop is released, and falls through the air, impacting and bouncing off the bottom wall. The objective of this problem is to simulate and study the behavior of the water drop.

The physical properties of the air, water and the air-water interface employed in this problem are listed in Table 4. The air and the water are respectively assigned as the first and the second fluids in the simulations. We use *L* as the characteristic length scale, and choose the characteristic velocity scale $U_{0}=\sqrt{g_{r0}L}$, where *g*_*r*0_ = 1*m*/*s*^2^. The problem is then non-dimensionalized according to Table 1.

**Table 4:** Physical properties of air and water.

Density [*kg*/*m*^3^]	air: 1.2041	water: 998.207
Dynamic viscosity [*kg*/(*m* ⋅ *s*)]	air: 1.78 × 10^−5^	water: 1.002 × 10^−3^
Surface tension [*kg*/*s*^2^]	air-water: 7.28 × 10^−2^	
Gravity [*m*/*s*^2^]	9.8	

To simulate the problem we discretize the domain using 150 equal-sized quadrilateral elements, with 10 and 15 elements in the *x* and *y* directions respectively. We use an element order 14 for all elements in the simulations. The algorithm developed here is employed for marching in time. In the horizontal direction we employ periodic boundary conditions for all flow variables. At the top and the bottom walls, we impose the velocity Dirichlet condition Eq (8) with **w** = 0, and impose the contact-angle boundary conditions Eqs (10a) and (10b) with *g*_*c*1_ = *g*_*c*2_ = 0 and *θ*_*s*_ = 10° for the phase field function. Note that *θ*_*s*_ in Eq (10b) is the angle measured on the side of the first fluid, that is, the air for the current configuration. We employ the following initial velocity and phase field function distributions, **u**_*in*_ = 0 and $\phi_{in}=\tanh\frac{∥x-X_{0}∥-R_{0}}{\sqrt{2}\eta },$ where **X**_0_ = (*x*_0_, *y*_0_) is the initial coordinate of the drop center of mass. Two different domain sizes with *L* = 4mm and *L* = 2mm have been considered in the simulations, corresponding to two drop radii *R*_0_ = 1mm and *R*_0_ = 0.5mm, respectively. The majority of simulations are performed with the drop radius 1mm. The physical/numerical parameter values are summarized in Table 5.

**Table 5:** Physical/numerical parameters for the bouncing water drop problem.

parameters	values	parameters	values
*ρ*_2_/*ρ*_1_	829.01	*ρ*_*m*_	min(*ρ*_1_, *ρ*_2_)
*μ*_2_/*μ*_1_	56.29	*ν*_*m*_	$\frac{1}{2}(\frac{\mu_{1}}{\rho_{1}}+\frac{\mu_{2}}{\rho_{2}})$
*L*	4mm or 2mm	Δ*tU*_0_/*L*	2.5 × 10^−5^ (*L* = 4mm)
*μ*_1_/(*ρ*_1_ *U*_0_ *L*)	5.843 × 10^−2^ (*L* = 4mm)		1.0 × 10^−5^ (*L* = 2mm)
	1.653 × 10^−1^ (*L* = 2mm)	$\lambda /(\rho_{1}U_{0}^{2}L^{2})$	$\frac{3}{2\sqrt{2}}\frac{\sigma }{\rho_{1}U_{0}^{2}L}\frac{\eta }{L}$
*η*/*L*	0.01	$g_{r}L/U_{0}^{2}$	9.8
$\sigma /(\rho_{1}U_{0}^{2}L)$	3778.76 (for *L* = 4mm)	(*γ*_1_ *ρ*_1_ *U*_0_)/*L*	${\left(\frac{\eta }{L}\right)}^{3}\frac{1}{\lambda /(\rho_{1}U_{0}^{2}L^{2})}$
	15115 (for *L* = 2mm)	*θ*_*s*_	10°
Elements	150	Element order	14
*H*_0_	(varied)	*J*	2

Let us first look into the dynamics of this air-water two-phase system. Fig 4 shows a temporal sequence of snapshots of the air-water interface. This corresponds to the water drop radius 1mm, and an initial drop height *H*_0_ = 3.2mm above the bottom wall. The air-water interface is visualized by the contour levels *ϕ* = 0 at different time instants. Upon release, the water drop falls through the air (Fig 4(a) and 4(b)), and impacts the bottom wall (Fig 4(c)). One can observe a notable deformation of the water drop upon impact of the wall. Subsequently, the water drop bounces off the bottom wall (Fig 4(d)) and rises through the air, reaching a maximum height (Fig 4(e)). Then the drop falls through the air again and impacts the bottom wall a second time (Fig 4(f)–4(h)). This process repeats several times, and the water drop eventually settles down on the bottom wall.

**Fig 5:**
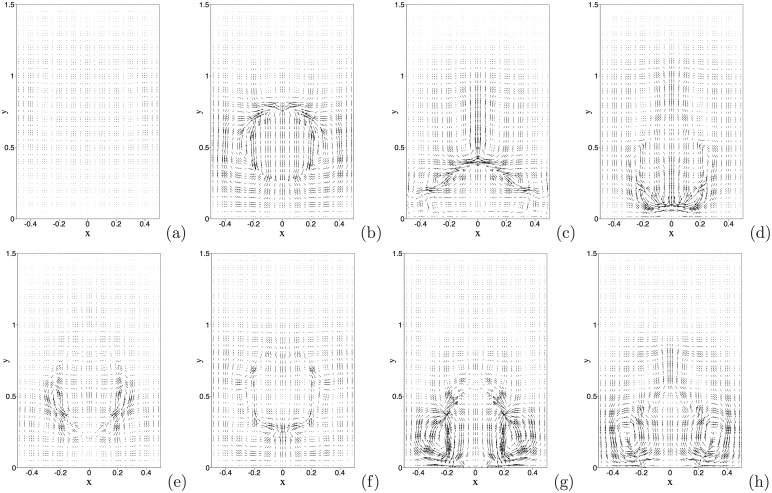
Bouncing water drop (drop radius 1mm, initial height *H*_0_ = 3.2mm): temporal sequence of snapshots of the velocity field at non-dimensional time instants: (a) *t* = 0.05, (b) *t* = 0.25, (c) *t* = 0.4, (d) *t* = 0.55, (e) *t* = 0.7, (f) *t* = 0.85, (g) *t* = 1.0, (h) *t* = 1.15. Velocity vectors are plotted on every fifth quadrature points in each direction within each element.

Fig 5 is a temporal sequence of snapshots of the velocity fields of this system at the same time instants as those of Fig 4. The interaction between the air and water, and the impact of the water drop on the wall, induce complicated velocity patterns in the domain. It can be observed that the motion of the water drop has induced a velocity field in the air (see Fig 5(b), 5(e) and 5(f)). As the water drop impacts the wall, it can be discerned from the velocity patterns that the air is expelled from the near-wall region; see Fig 5(g) and 5(c). As the water drop bounces off or is about to bounce off, the velocity distribution indicates that the air rushes from aside to fill in the near-wall region; see Fig 5(d) and 5(h).

**Fig 6:**
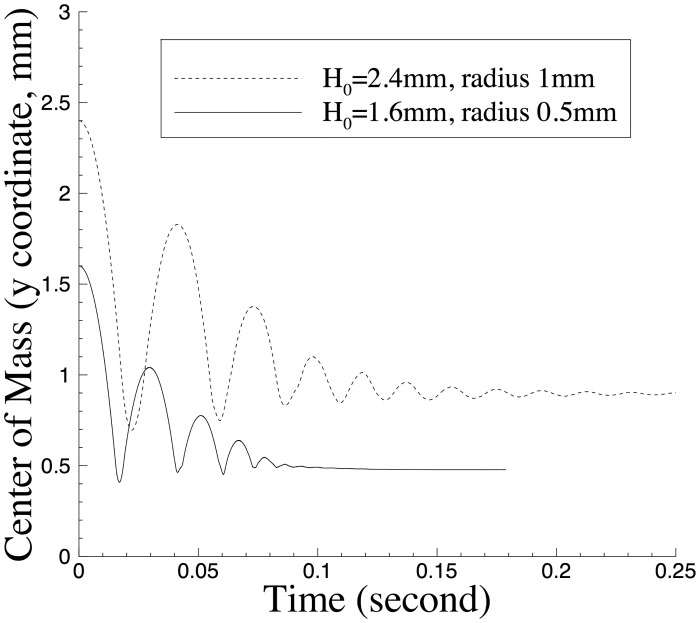
Time histories of the water-drop center of mass (y coordinate).

We have monitored the motion of the drop center of mass in simulations. The drop center of mass is defined by $X_{w}=\left(x_{w},y_{w}\right)=\frac{\int_{\Omega_{w}}xdx}{\int_{\Omega_{w}}dx},$ where Ω_*w*_(*t*) is the domain occupied by the water drop at time *t* and demarcated by the contour level *ϕ* = 0. In Fig 6 we show the time histories of the *y* coordinate of the drop center of mass for two cases with different drop radius. It can be discerned that the water drop bounces off the bottom wall a number of times in both cases. One can also discern an oscillation in the drop shape in later time with the larger water drop, before it completely settles down on the wall.

We have computed the restitution coefficient based on the time histories of the center of mass. We follow [39] and define the restitution coefficient by $C_{res}=\sqrt{\frac{H^{\prime }}{H}},$ where *H* and *H*′ respectively denote the drop maximum heights above the bottom wall before and after the bounce. We again follow [39] and estimate the impact velocity of the water drop by $V_{imp}=\sqrt{2g_{r}H},$ where *g*_*r*_ is the gravitational acceleration.

**Fig 7:**
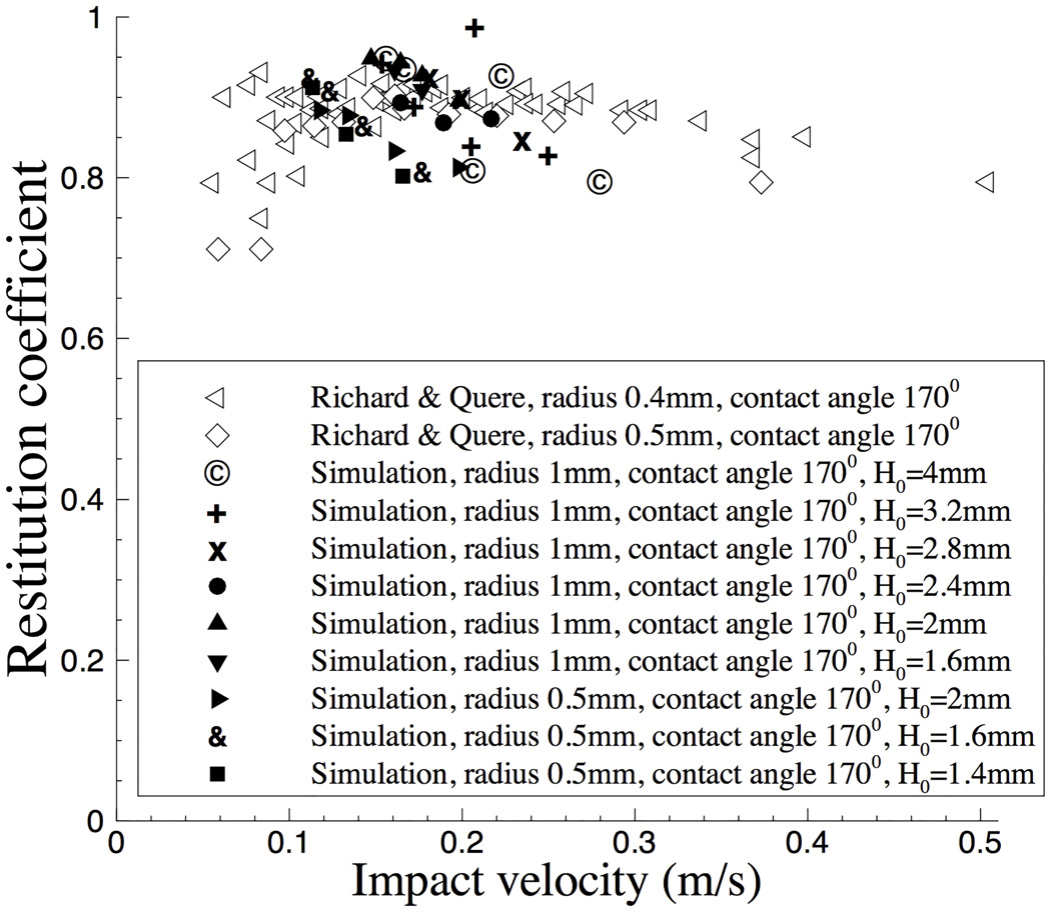
Comparison of restitution coefficient as a function of impact velocity between current simulations and the experiment [39].

In Fig 7 we plot the restitution coefficient *C*_*res*_ versus the impact velocity *V*_*imp*_ from the current simulations. For comparison, we also show the restitution coefficient data from the experiment of [39]. The restitution coefficients corresponding to the two drop radii and different initial drop heights *H*_0_ from the simulations have been included in this figure. The bulk of the restitution coefficients from the current simulations appear to agree quite well with the experimentally determined values. On the other hand, some differences can also be observed, especially for the data points corresponding to the first couple of bounces with larger initial drop-height values. We observe that for such cases the restitution coefficients from the simulation tend to be a little smaller than the bulk of the experimental values. This is likely due to the larger drop deformation upon impact, associated with a larger initial drop height and a larger impact velocity. The elastic energy associated with the drop deformation may reduce the maximum height the drop can reach after the bounce, and thus results in a smaller restitution coefficient. We have also observed that in some occasional case the water drop with radius 1mm can reach a maximum height after a bounce that is quite close to that before the bounce; see the outlying data point with a large restitution coefficient (symbol “+”), which corresponds to an initial drop height *H*_0_ = 3.2mm. The larger bounce-off height is possibly due to the conversion of the elastic energy associated with the drop-shape oscillation before the bounce into the kinetic energy associated with the drop center of mass after the bounce and lift-off.

The above comparison indicates that the simulation results obtained using our method overall agree reasonably well with the experimental measurement.

### Air Jet in Water with Two-Phase Open Boundaries

The goal of this section is to demonstrate the effectiveness of the open boundary conditions and our algorithm for two-phase outflow problems. The test problem considered here involves open boundaries where the two fluids may leave or enter the domain, large density contrast, and large viscosity contrast. The fluid interface passes through the open domain boundary in this problem.

We consider the long-time behavior of an air-water two-phase flow, in which a train of air bubbles continually forms at a wall inside the water and then moves out of the domain due to buoyancy. This flow problem has been considered in a previous work [6]. It should be noted that the open boundary conditions and the numerical algorithm being tested here are different.

**Fig 8:**
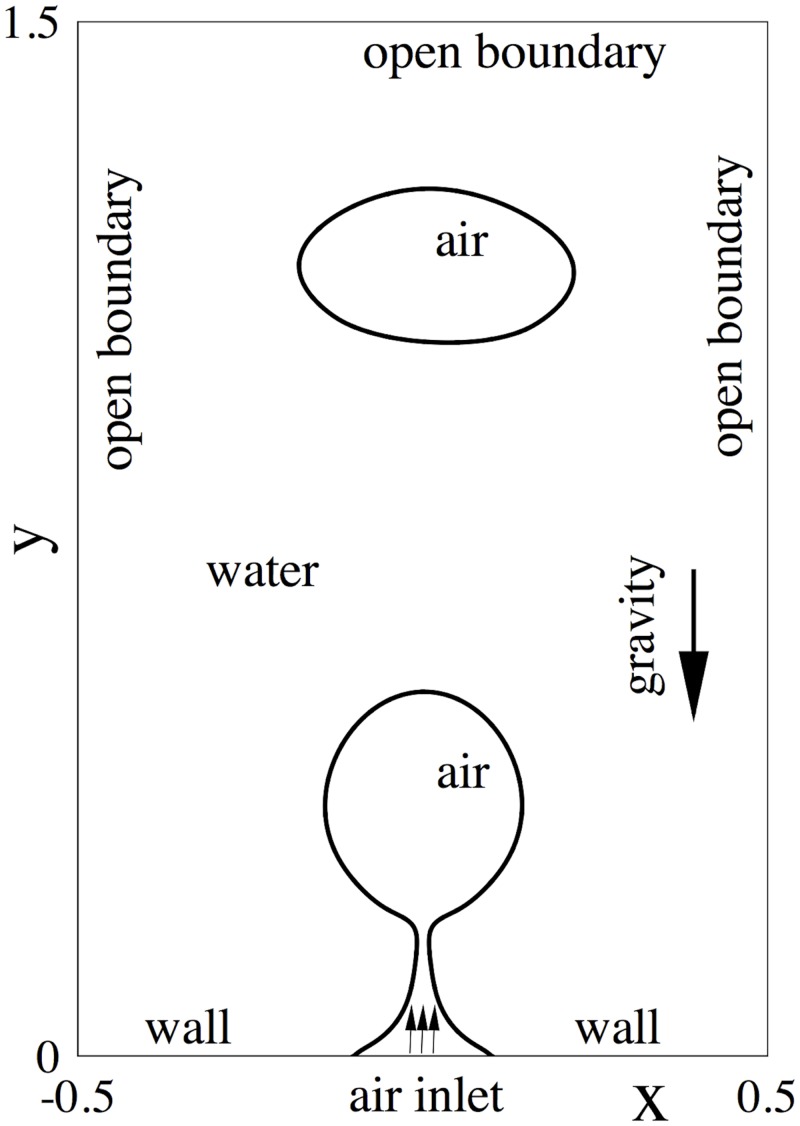
Configuration of the air jet in water problem.

Specifically, we consider the flow domain shown in Fig 8, $-\frac{L}{2}⩽x⩽\frac{L}{2}$ and $0⩽y⩽\frac{3L}{2}$, where *L* = 3cm. The bottom of the domain is a solid wall, while the other three sides (top, left and right) are all open, where the fluid can freely leave or enter the domain. The domain is initially filled with water, and the gravity is along the vertical direction pointing downward. The bottom wall has an orifice in its center, with a diameter *d* = 6mm. A stream of air is continuously injected into the domain through the orifice. The air velocity has a parabolic profile at the orifice, with a centerline value *U*_0_ = 17.3*cm*/*s*. The bottom wall has a neutral wettability, that is, if the air-water interface intersects the wall the contact angle would be 90°. Our objective is to simulate and study the long-time behavior of this system.

The physical parameters concerning the air, water and the air-water interface have been provided in Table 4. We treat the air and the water as the first and the second fluids, respectively. *L* and *U*_0_ are employed respectively as the characteristic length and velocity scales. Normalization of the problem then proceeds according to Table 1.

The flow domain is discretized using 600 quadrilateral spectral elements, with 20 and 30 elements in the *x* and *y* directions respectively. An element order 12 has been used for all elements in the simulations. At the bottom wall, we impose the velocity Dirichlet condition Eq (8) with **w** = 0 and the boundary conditions Eqs (10a) and (10b) with *g*_*c*1_ = *g*_*c*2_ = 0 and *θ*_*s*_ = 90°. At the air inlet we impose the velocity Dirichlet condition Eq (8), in which **w** has zero horizontal component and its vertical component takes a parabolic profile with a centerline value *U*_0_; for the phase field function, we impose the boundary conditions Eqs (9a) and (9b), in which *g*_*b*_ = 0 and

$$\begin{array}{c} \phi_{b}\left(x,t\right)=-\tanh\frac{x-R}{\sqrt{2}\eta }\left[H(x,0)-H(x,R)\right]+\tanh\frac{x+R}{\sqrt{2}\eta }\left[H(x,-R)-H(x,0)\right] \end{array}$$

where $R=\frac{d}{2}=3mm$ is the radius of the orifice, and *H*(*x*, *a*) is the heaviside step function taking unit value if *x* ⩾ *a* and vanishing otherwise. On the top, left and right sides of the domain, we impose the open boundary condition Eq (13) with **f**_*b*_ = 0 for the momentum equation; for the phase field function we impose the boundary conditions Eqs (7a) and (7b) with *g*_*a*1_ = *g*_*a*2_ = 0. For the initial conditions, we have used an instantaneous snapshot of the velocity field and the phase field function from the simulation of [6]. Because long-time simulations have been performed, the initial velocity and phase field distributions have no effect on the long-time behavior of the system.

We apply an external pressure gradient in the *y* direction ($-\frac{\Delta P}{L}$) to balance the weight of water in the simulations, i.e. $-\frac{\Delta P}{L}=\rho_{w}g_{r},$ where *ρ*_*w*_ is the water density and *g*_*r*_ is the magnitude of the gravitational acceleration.

Table 6 summarizes the physical/numerical parameter values for this problem. The *D*_0_ in the open boundary condition Eq (7b) for the phase field function is determined based on a preliminary simulation with *D*_0_ = 0. Preliminary simulations indicate that the air bubbles have a non-dimensional convection velocity about 2.0 ∼ 3.0 at the upper domain boundary. Because $\frac{1}{D_{0}}$ plays the role of a convection velocity, we therefore use an outflow dynamic mobility $\frac{1}{D_{0}U_{0}}\approx 2.5$ in the simulations.

**Table 6:** Physical and numerical parameter values for the air jet in water problem.

parameters	values	parameters	values
*ρ*_2_/*ρ*_1_	829.01	*ρ*_*m*_	min(*ρ*_1_, *ρ*_2_)
*μ*_1_/(*ρ*_1_ *U*_0_ *L*)	2.845 × 10^−2^	*ν*_*m*_	$50\;\max(\frac{\mu_{1}}{\rho_{1}},\frac{\mu_{2}}{\rho_{2}})$
*μ*_2_/*μ*_1_	56.29	Δ*tU*_0_/*L*	1.5 × 10^−6^
*η*/*L*	0.01	*θ*_*s*_	90°
$\sigma /(\rho_{1}U_{0}^{2}L)$	67.178	(*γ*_1_ *ρ*_1_ *U*_0_)/*L*	$0.1{\left(\frac{\eta }{L}\right)}^{3}\frac{1}{\lambda /(\rho_{1}U_{0}^{2}L^{2})}$
$\lambda /(\rho_{1}U_{0}^{2}L^{2})$	$\frac{3}{2\sqrt{2}}\frac{\sigma }{\rho_{1}U_{0}^{2}L}\frac{\eta }{L}$	$g_{r}L/U_{0}^{2}$ (gravity)	9.8
*D*_0_ *U*_0_	0.4	*μ*_0_	20 max(*μ*_1_, *μ*_2_)
Number of elements	600	Element order	12
−Δ*P*/*L*	*ρ*_*w*_ *g*_*r*_	*J*	2
*δ*	0.01		

**Fig 9:**
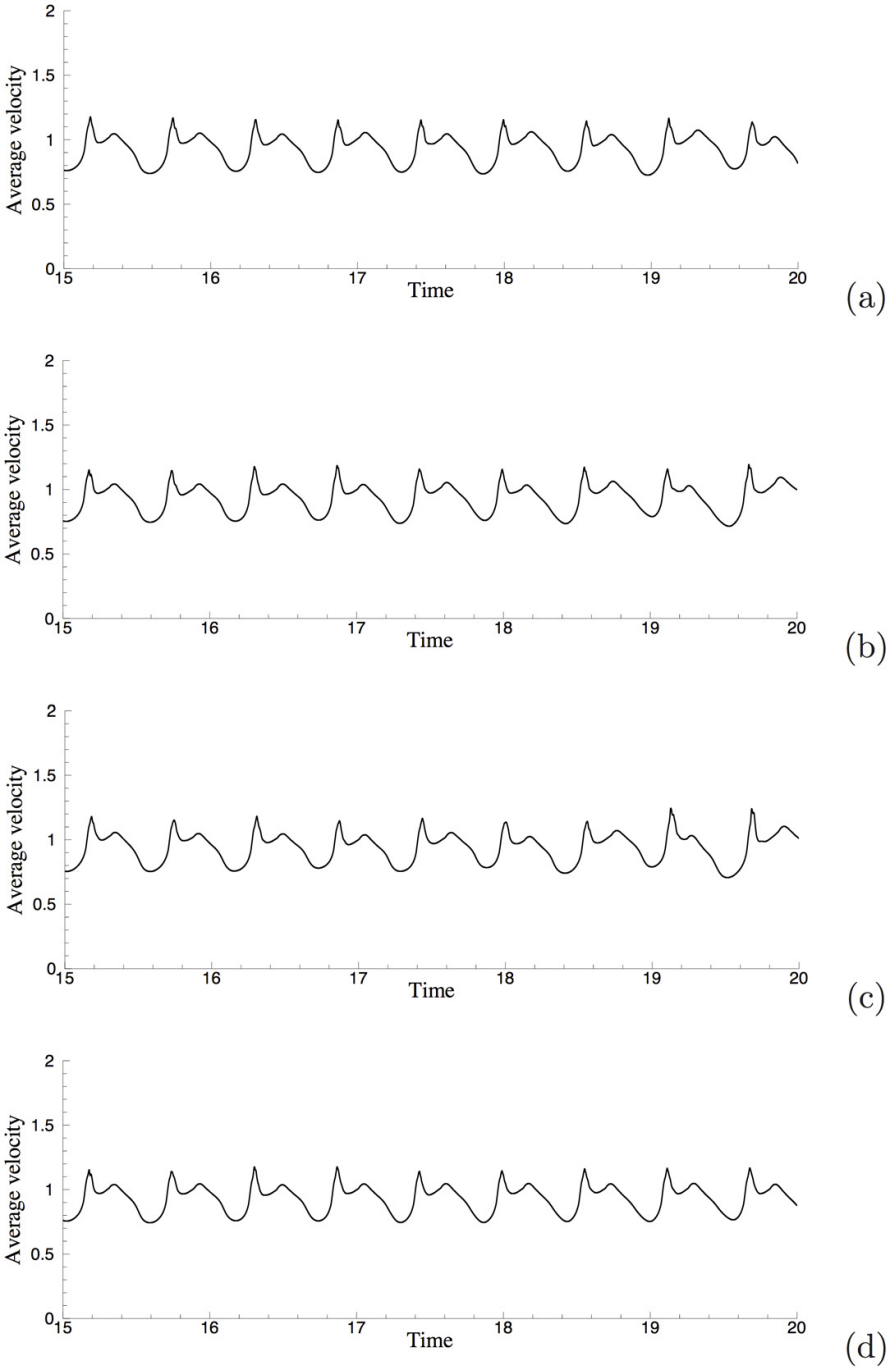
Time histories of average vertical-velocity magnitude from different open boundary conditions: (a) OBC Eq (3a), (b) OBC Eq (3b), (c) OBC Eq (3c), (d) OBC Eq (3d).

Let us first demonstrate the long-term stability of the computation. We have performed long-time simulations of this problem using different open boundary conditions. Fig 9 shows a window of the time histories of the average vertical velocity magnitude, $V_{\text{avg}}\left(t\right)={\left(\frac{1}{V_{\Omega }}\int_{\Omega }{\left|v\right|}^{2}d\Omega \right)}^{\frac{1}{2}},$ where *v* is the *y* velocity component and *V*_Ω_ = ∫_Ω_ *dΩ* is the volume of the domain. Results in Fig 9(a)–9(d) are obtained using the open boundary conditions Eqs (3a)–(3d), respectively. One can make two observations. First, the average velocity magnitude *V*_avg_ fluctuates over time about some constant mean level and its time history signal exhibits a quasi-periodic nature. This indicates that the flow is at a statistically stationary state, and that the computations using our algorithm and the several outflow boundary conditions are stable over a long time. Second, the time-history curves obtained with different open boundary conditions Eqs (3a) and (3b) are qualitatively similar, indicating that these boundary conditions lead to similar results about the flow.

**Fig 10:**
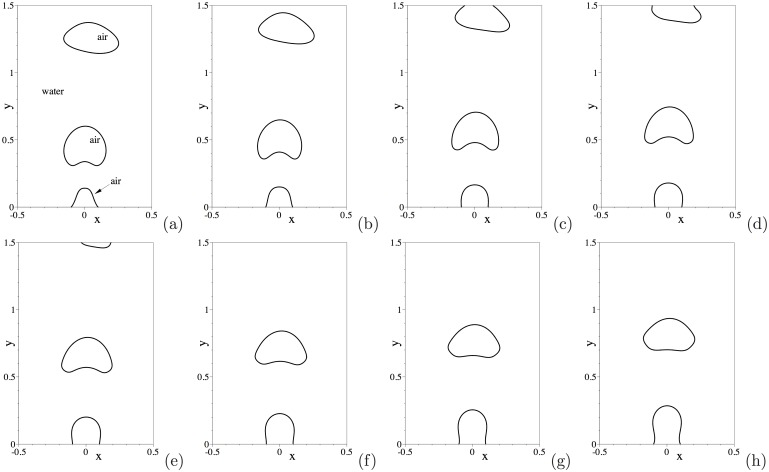
Air jet in water: temporal sequence of snapshots of the air-water interface at time instants (a) *t* = 16.9397, (b) *t* = 16.9772, (c) *t* = 17.0222, (d) *t* = 17.0522, (e) *t* = 17.0897, (f) *t* = 17.1272, (g) *t* = 17.1647, (h) *t* = 17.2022. Results are obtained using the boundary condition Eq (3b) on the open boundaries.

The dynamics of this air-water flow is illustrated by Fig 10, in which we show a temporal sequence of snapshots of the air-water interface in a time-window between *t* = 16.9397 and *t* = 17.2022. The fluid interface is visualized using the contour level *ϕ*(**x**, *t*) = 0 in the plots. These results are obtained with the open boundary condition Eq (3b), corresponding to the time history in Fig 9(b). These plots demonstrate the process of free air bubbles generated at the wall rising through water and crossing the upper domain boundary to migrate out of the domain. Fig 10(a)–10(e) shows the leading air bubble passing through the upper open boundary of the domain. They demonstrate that our method can effectively allow the fluid interface to pass through the open/outflow boundary in a smooth fashion. Simultaneously, one can observe that the trailing free bubble rises through the water, and that a new air bubble is forming at the bottom wall (Fig 10(b)–10(h)). Subsequently, the air bubble at the wall breaks free, and the above process will repeat itself.

**Fig 11:**
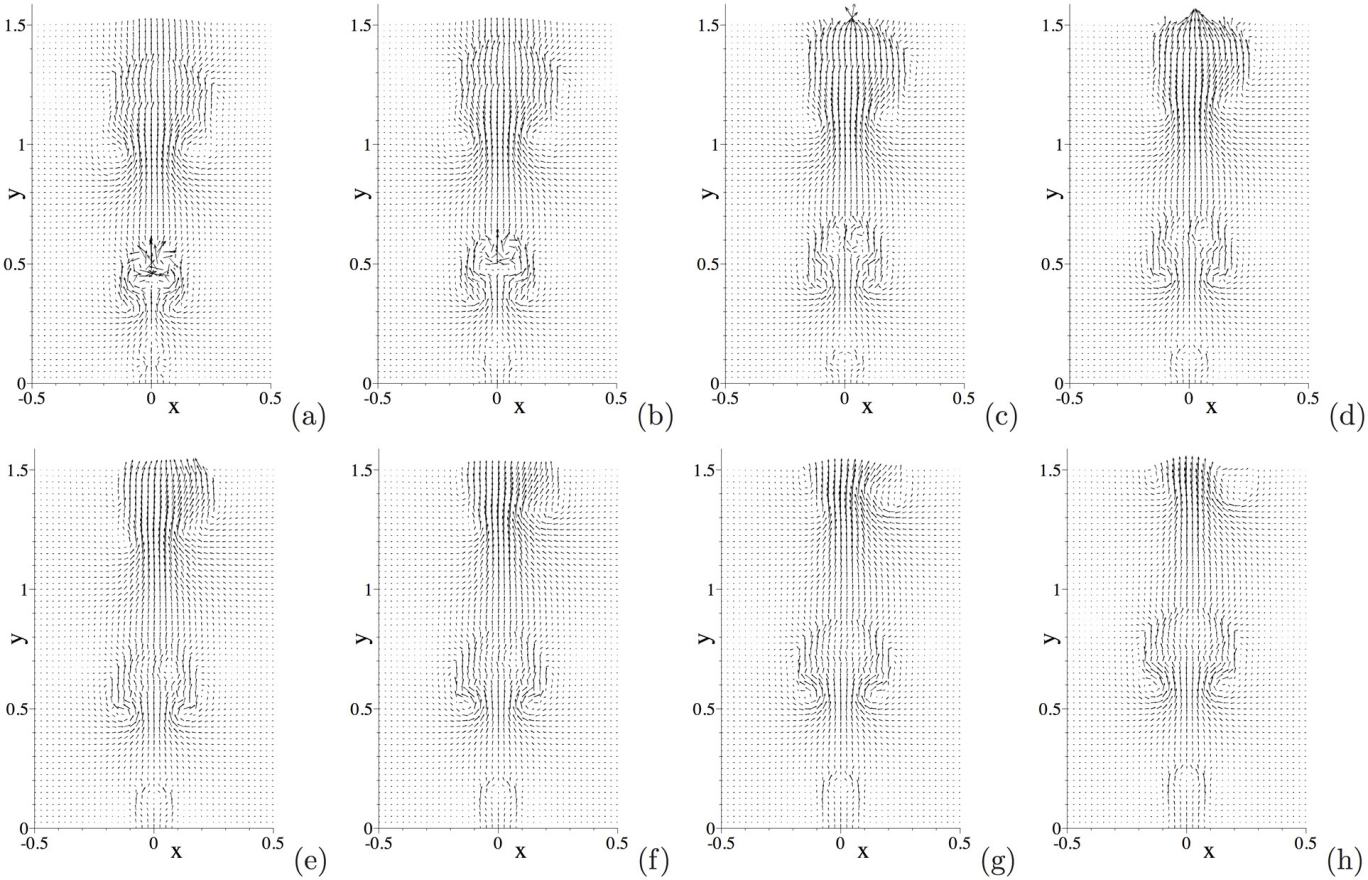
Air jet in water: temporal sequence of snapshots of the velocity field at time instants (a) *t* = 16.9397, (b) *t* = 16.9772, (c) *t* = 17.0222, (d) *t* = 17.0522, (e) *t* = 17.0897, (f) *t* = 17.1272, (g) *t* = 17.1647, (h) *t* = 17.2022. Velocity vectors are plotted on every ninth quadrature points in each direction within each element. Results are obtained using the boundary condition Eq (3b) on the open boundaries.

We further illustrate the flow dynamics using instantaneous velocity distributions. Fig 11 is a temporal sequence of snapshots of the velocity fields at identical time instants as those of the interfacial plots of Fig 10. One can observe that a significant flow field is induced in the regions occupied by the air bubbles, and that a particularly strong velocity field exists inside the free air bubble as it initially breaks free from the wall; see the region of the trailing free bubble in Fig 11(a) and 11(b). On the other hand, the velocity field in the water region is in general quite weak. As the air bubble rises through the water, a pair of vortices forms in the water region trailing the air bubble; see the region behind the second air bubble in Fig 11(e)–11(h). These vortices can induce a backflow on portions of the outflow/open boundary after the air bubble passes through (Fig 11(h)).

One can notice that as the air bubble moves farther downstream of the inlet its shape loses symmetry with respect to the centerline of the domain and its path seems to deviate from the centerline (Fig 10(a)–10(d)), and that the velocity field also becomes non-symmetric away from the inlet (Fig 11). The loss of symmetry and the path instability of air bubbles in water depend on the bubble size and the Reynolds number, and have been well documented in the literature; see e.g. [40–43]

**Fig 12:**
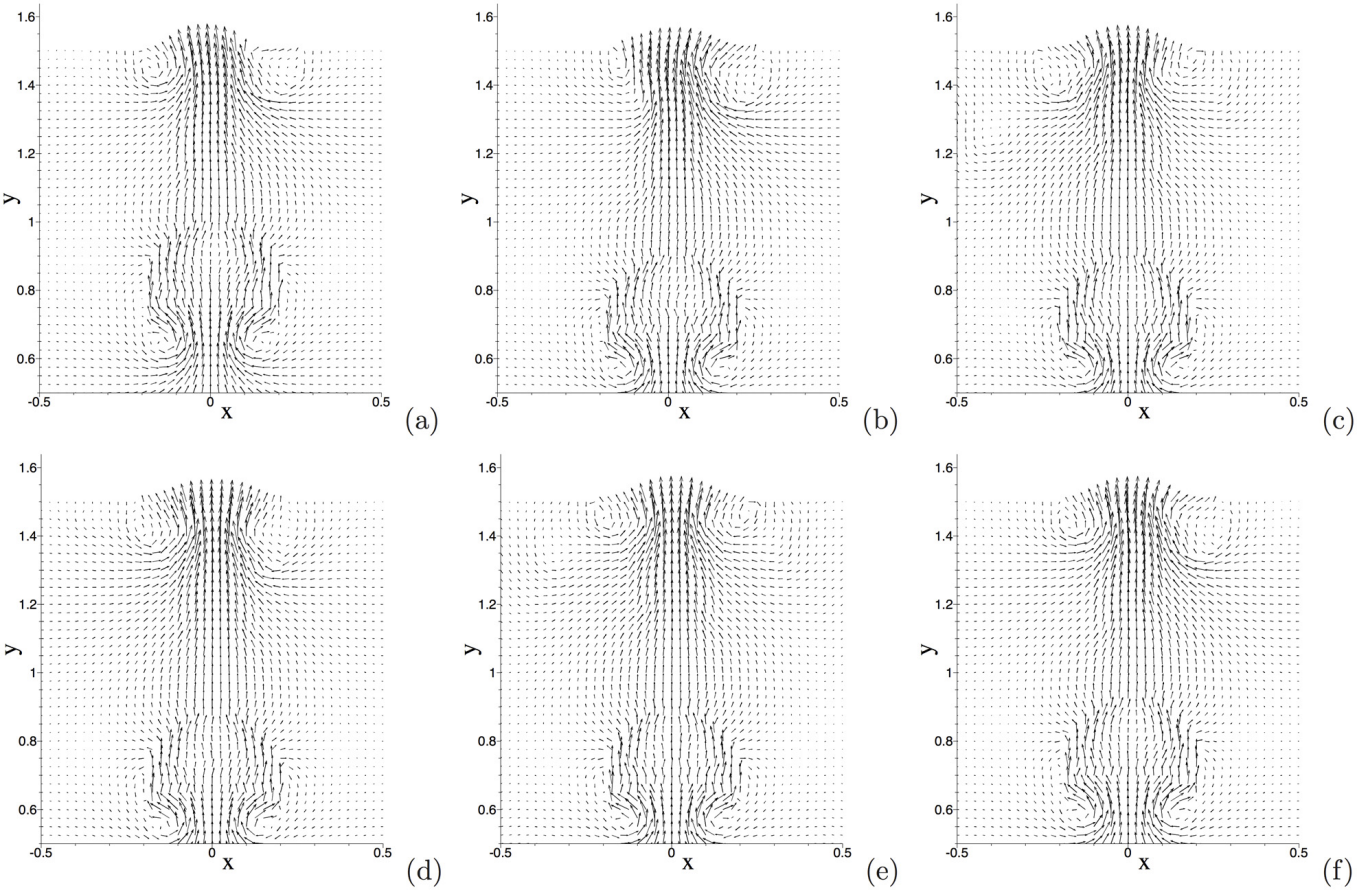
Velocity distributions near the upper domain boundary obtained with various open boundary conditions: (a) OBC Eq (3a), (b) OBC Eq (3b), (c) OBC Eq (3c), (d) OBC Eq (3d), (e) OBC Eq (4), (f) OBC Eq (5). Velocity vectors are plotted on every ninth quadrature points in each direction within each element.

We observe that the open boundary conditions Eqs (3a)–(3d) proposed here produce flow characteristics that are similar to those based on the boundary conditions Eqs (4) and (5) from [6]. A comparison of the velocity distributions near the upper domain boundary obtained with different open boundary conditions is shown in Fig 12. This corresponds to a configuration where the pair of vortices trailing a free air bubble crosses the upper open boundary. Only the upper portion of the domain has been shown here for clarity. One can clearly observe the backflows into the domain induced by the vortices on sections of the upper boundary. Flows are also sucked into the domain through the side boundaries. The overall characteristics of the velocity fields obtained with these open boundary conditions appear qualitatively quite similar.

**Fig 13:**
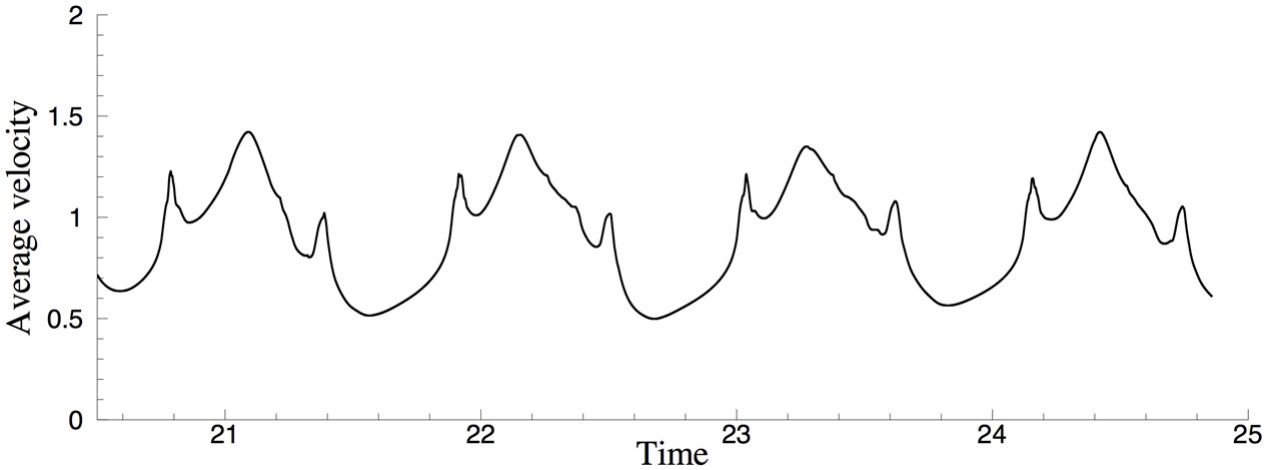
Another window of time history of the average vertical-velocity magnitude, suggesting a somewhat different flow state. Result is obtained using the open boundary condition Eq (3b).

The results presented so far illustrate one state of the flow. We observe that this air-water flow can exhibit another state, in which the flow characteristics are somewhat different than those seen above. In Fig 13 we show another window in the time history of the average vertical velocity magnitude, obtained with the open boundary condition Eq (3b). The flow evidently is at a statistically stationary state. Contrasting this figure with Fig 9(b), which is computed using the same boundary conditions, we can observe that the velocity-history curves have qualitatively different characteristics.

**Fig 14:**
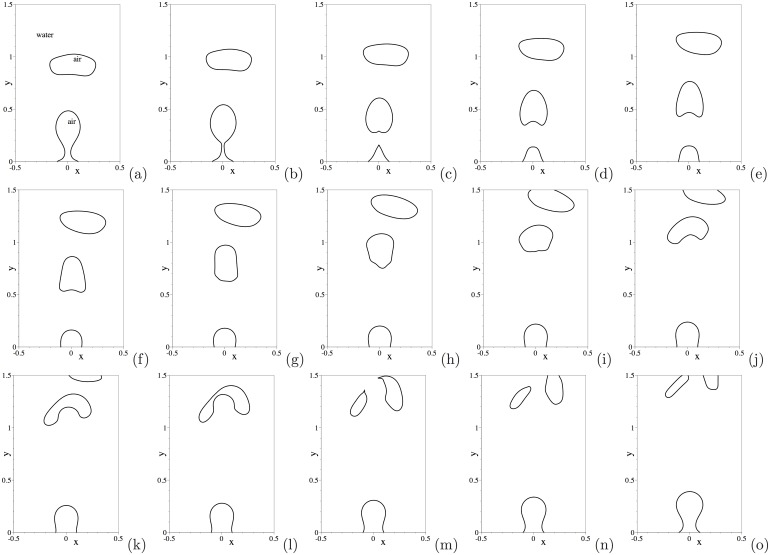
Air jet in water: Temporal sequence of snapshots of the air-water interface at time: (a) *t* = 22.9997, (b) *t* = 23.0372, (c) *t* = 23.0747, (d) *t* = 23.1122, (e) *t* = 23.1497, (f) *t* = 23.1872, (g) *t* = 23.2247, (h) *t* = 23.2622, (i) *t* = 23,2922, (j) *t* = 23.3222, (k) *t* = 23.3522, (l) *t* = 23.3822, (m) *t* = 23.4197, (n) *t* = 23.4572, (o) *t* = 23.5172. Results are obtained using the open boundary condition Eq (3b).

This different flow state is further illustrated by the temporal sequence of snapshots of the air-water interface shown in Fig 14, which covers a time window between *t* ≈ 23 and *t* ≈ 23.5 in the history plot of Fig 13. These results correspond to the open boundary condition Eq (3b). The plots clearly show the breakaway of the air bubble from the wall (Fig 14(a)–14(c)) and the bubble motion across the domain and the upper open boundary (Fig 14(d)–14(k)). The crucial difference, when compared with Fig 10, lies in the following. When multiple free bubbles are present in the domain, the interaction between the leading-bubble wake and the trailing bubble appears to have caused the trailing bubble to accelerate and nearly cath up with the leading one; see Fig 14(e)–14(j). This has also induced significant deformations in the trailing bubble (Fig 14(j)–14(l)), and caused it to subsequently break up (Fig 14(m)–14(o)). As the free bubbles (and their daughter bubbles) quickly move out of the domain, one can observe that another bubble is forming, but still attached to the wall (Fig 14(o)). Consequently, the flow domain will be depleted of free bubbles for a period of time beyond the time instant corresponding to Fig 14(o), until the air bubble attached to the wall breaks free. This scenario is more similar to the one discussed in [6], but is quite different from that shown by Fig 10. From Fig 14(i)–14(k) we can again observe that our method allows the air bubble and the air-water interface to cross the open boundary in a smooth fashion.

**Fig 15:**
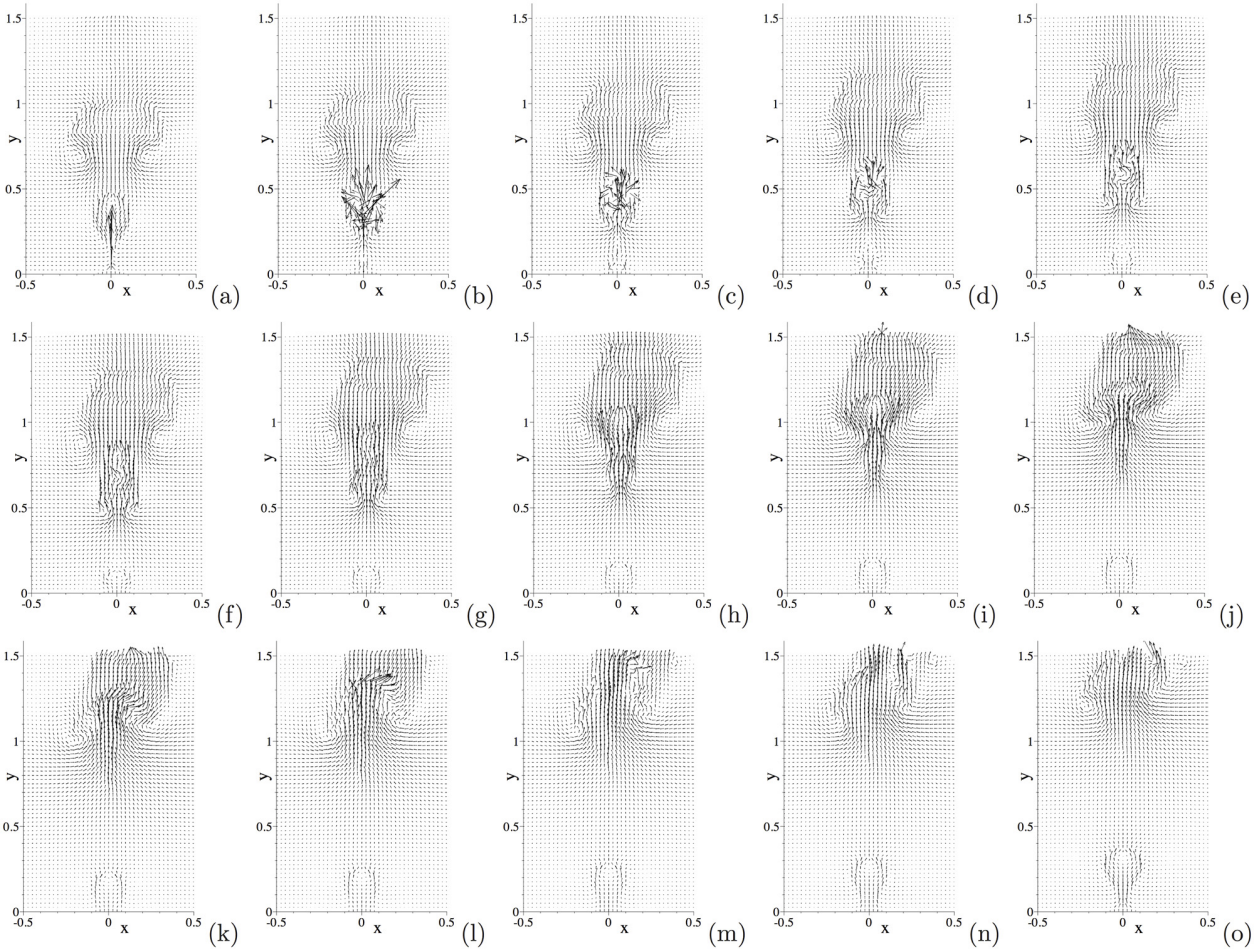
Air jet in water: Temporal sequence of snapshots of the velocity field at time: (a) *t* = 22.9997, (b) *t* = 23.0372, (c) *t* = 23.0747, (d) *t* = 23.1122, (e) *t* = 23.1497, (f) *t* = 23.1872, (g) *t* = 23.2247, (h) *t* = 23.2622, (i) *t* = 23,2922, (j) *t* = 23.3222, (k) *t* = 23.3522, (l) *t* = 23.3822, (m) *t* = 23.4197, (n) *t* = 23.4572, (o) *t* = 23.5172. Velocity vectors are plotted on every ninth quadrature point in each direction within each element. Results are obtained using the open boundary condition Eq (3b).

Fig 15 shows the corresponding velocity distributions at the same time instants as those of Fig 14. One can observe the intense velocity field inside the air bubble shortly before/after it breaks free from the bottom wall (Fig 15(a)–15(d)). The pair of vortices formed behind the leading free bubble is evident from Fig 15(a)–15(f). The flow induced by this vortex pair causes the trailing free bubble to squeeze through the vortex pair. This can be discerned from Fig 15(g)–15(j) and Fig 14(g)–14(j). Comparison between Figs 15(k) and 14(k) indicates that the trailing bubble is situated above the vortex pair at that instant. Subsequently, the intense upward flow induced by the vortex pair causes the bubble to deform severely and break up into two smaller bubbles; see Fig 14(k)–14(n) and Fig 15(k)–15(n).

The air jet in water problem is a stringent test to the open boundary conditions. The presence of two-phase open boundary, combined with the large density ratio between air and water, makes this problem extremely challenging to simulate. The results of this section show that the two-phase open boundary conditions and the numerical algorithm developed in the current work are effective for two-phase outflows with large density and viscosity contrasts at the outflow boundaries. The **E**(*ρ*, **n**, **u**) term in the open boundary condition Eq (13) is critical to the stability for this problem. We observe that the computation using an open boundary condition without this term is unstable for this problem, that is,

$$\begin{array}{c} -pn+\mu n\cdot D\left(u\right)-\left[\frac{\lambda }{2}\nabla \phi \cdot \nabla \phi +F\left(\phi \right)\right]n=0,\;\text{on}\;\partial \Omega_{o}, \end{array}$$ (35)

due to the backflows induced by the vortices at the outflow boundary. It is observed that increasing *ν*_*m*_ in the algorithm tends to improve the stability, and that a larger *μ*_0_ in Eq (20c) for the numerical treatment of the open boundary condition also improves the stability for the current pressure-correction based scheme. This observation concerning *μ*_0_ seems different from the trend observed in [6], which is for a velocity-correction based algorithm.

## Concluding Remarks

We have presented several new open boundary conditions for two-phase outflows, and a rotational pressure-correction based algorithm for solving the two-phase momentum equations together with these boundary conditions. These techniques are then combined with a solver for the phase-field equation to form an efficient and effective method for incompressible two-phase flows involving open/outflow boundaries.

The two-phase open boundary conditions developed herein are inspired by the the general form of *single-phase* open boundary conditions from [5] and the two-phase energy balance discussed in [6]. The current work provides several new forms of two-phase open boundary conditions beyond those developed in [6].

The algorithm presented herein for the two-phase momentum equations is based on a rotational pressure correction-type strategy for de-coupling the velocity/pressure computations. More importantly, the current algorithm results in velocity and the pressure linear algebraic systems with *constant* and *time-independent* coefficient matrices after discretization, despite the variable nature of the mixture density and mixture viscosity. Therefore, these coefficient matrices can be pre-computed during pre-processing. In a previous work [13] we have developed a *velocity correction-based* algorithm for the variable-density Navier-Stokes equations that possesses similar properties (leading to constant coefficient matrices for pressure/velocity linear systems); see also subsequent applications and further developments based on that algorithm in [6, 25, 26, 28]. The algorithm developed herein in a sense is the *pressure-correction counterpart* to the scheme of [13]. The implementation presented herein is suitable for *C*^0^ spectral elements, and with no change also applies to conventional finite elements.

The numerical treatments for the open boundary conditions proposed herein involve imposing a discrete Neumann type condition on the outflow boundary at the velocity substep, and two discrete Dirichlet type conditions on the outflow boundary at the substeps for *ξ*^*n*+1^ and pressure respectively. The discrete velocity-Neumann and the pressure-Dirichlet conditions on the outflow boundary stem largely from the continuous open boundary condition. But they contain modifications and additional terms that are essential to the stability of the algorithm.

To demonstrate the physical accuracy of the method developed herein, we have considered the capillary wave problem and compared quantitatively the numerical solution with the two-phase exact physical solution by [38] for a range of density ratios (up to 1000). The comparisons show that our method produces physically accurate results. We have also considered the bounce of a water droplet on a superhydrophobic surface, and compared the restitution coefficients from the simulations and the experimental measurement of [39]. The restitution coefficient values from both the experiment and the simulation exhibit a spread in a range. The bulk of the values from the simulations appear to agree well with those from the experiment. Some differences have also been observed, for those corresponding to the first couple of bounces with larger initial drop heights. In such cases, the simulation tends to produce restitution coefficient values that are close to but somewhat smaller than those from the experiment, likely due to the larger drop deformation upon impact associated with a larger initial drop height. These results lend confidence that the simulation has captured the flow characteristics reasonably well.

We have further simulated the air jet in water problem to test the effectiveness of the open boundary conditions and algorithm for two-phase problems with outflow/open boundaries. This problem involves large density ratio, large viscosity ratio, and backflows/vortices at the two-phase open boundary. The results demonstrate the long-time stability of our method. The method allows the fluid interface to pass through the open boundary in a smooth and seamless fashion.

Two-phase outflow/open boundary condition is an important and challenging issue in two-phase flow simulations, but it has been scarcely studied in the literature. Large contrasts in densities and viscosities and strong backflows/vortices at the outflow/open boundary present severe stability difficulties to two-phase simulations. Our contribution lies in that, the several open boundary conditions proposed herein, together with the one we developed in [6], provide a set of effective methods for simulating two-phase problems involving outflow/open boundaries. The advantage of these methods is that they can deal effectively with large viscosity contrast, large density contrast and strong vortices/backflows at the outflow/open boundaries. These methods allow for the simulation of long-time behaviors of two-phase flows so that statistically stationary states can be examined. We anticipate that they will be instrumental in the investigations of statistical characteristics of two-phase flows, where long-time sampling/averaging of the statistically stationary states will be required.

## Appendix: Algorithm for the Phase-Field Equation

This Appendix provides a summary of the algorithm we developed in [13] for solving the phase field Eq (1c). The notation here follows that of the main text.

Consider the system consisting of the phase field Eq (1c), inflow conditions Eqs (9a) and (9b), wall boundary conditions Eqs (10a) and (10b), and the open boundary conditions Eqs (7a) and (7b). Given $\left({\tilde{u}}^{n},\phi^{n}\right)$, where ${\tilde{u}}^{n}$ is the approximation velocity from the algorithm discussed in the main text, we discretize this system as follows:

$$\begin{array}{l} \frac{\gamma_{0}\phi^{n+1}-\hat{\phi }}{\Delta t}+{\tilde{u}}^{*,n+1}\cdot \nabla \phi^{*,n+1}\\ \;=-\lambda \gamma_{1}\nabla^{2}\left[\nabla^{2}\phi^{n+1}-\frac{S}{\eta^{2}}\left(\phi^{n+1}-\phi^{*,n+1}\right)-h\left(\phi^{*,n+1}\right)\right]+g^{n+1} \end{array}$$ (36a)

$$\begin{array}{cc} & \phi^{n+1}=\phi_{b}^{n+1},\;\text{on}\;\partial \Omega_{i} \end{array}$$ (36b)

$$\begin{array}{cc} & \nabla^{2}\phi^{n+1}-h\left(\phi^{n+1}\right)=g_{b}^{n+1},\;\text{on}\;\partial \Omega_{i} \end{array}$$ (36c)

$$\begin{array}{cc} & n\cdot \nabla \left[\nabla^{2}\phi^{n+1}-\frac{S}{\eta^{2}}\left(\phi^{n+1}-\phi^{*,n+1}\right)-h\left(\phi^{*,n+1}\right)\right]=g_{c1}^{n+1},\;\text{on}\;\partial \Omega_{w} \end{array}$$ (36d)

$$\begin{array}{cc} & n\cdot \nabla \phi^{n+1}=\frac{3\sigma }{4\lambda }\cos\theta_{s}\left[1-{\left(\phi^{*,n+1}\right)}^{2}\right]+g_{c2}^{n+1},\;\text{on}\;\partial \Omega_{w}. \end{array}$$ (36e)

$$\begin{array}{cc} & n\cdot \nabla \left[\nabla^{2}\phi^{n+1}-\frac{S}{\eta^{2}}\left(\phi^{n+1}-\phi^{*,n+1}\right)-h\left(\phi^{*,n+1}\right)\right]=g_{a1}^{n+1},\;\text{on}\;\partial \Omega_{o} \end{array}$$ (36f)

$$\begin{array}{c} n\cdot \nabla \phi^{n+1}=-D_{0}{\left.\frac{\partial \phi }{\partial t}\right|}^{*,n+1}+g_{a2}^{n+1},\;\text{on}\;\partial \Omega_{o} \end{array}$$ (36g)

$$\begin{array}{c} n\cdot \nabla \phi^{n+1}=-D_{0}\frac{\gamma_{0}\phi^{n+1}-\hat{\phi }}{\Delta t}+g_{a2}^{n+1},\;\text{on}\;\partial \Omega_{o}. \end{array}$$ (36h)

In the above equations, $\hat{\phi }$ is defined in Eq (24), ${\tilde{u}}^{*,n+1}$ and ϕ*^, *n*+1^ are defined in Eq (23), and *S* is a chosen constant that must satisfy the condition $S\;⩾\;n^{2}\sqrt{\frac{4\gamma_{0}}{\lambda \gamma_{1}\Delta t}}$. ${\frac{\partial \varphi }{\partial t}|}^{*,n+1}$ is an explicit approximation of $\frac{\partial \phi }{\partial t}$ at time step (*n* + 1), given by

$$\begin{array}{c} {\left.\frac{\partial \phi }{\partial t}\right|}^{*,n+1}=\left\{\begin{array}{cc} \frac{1}{\Delta t}\left(\phi^{n}-\phi^{n-1}\right), & \text{if}\;J=1\\ \frac{1}{\Delta t}\left(\frac{5}{2}\phi^{n}-4\phi^{n-1}+\frac{3}{2}\phi^{n-2}\right), & \text{if}\;J=2. \end{array}\right. \end{array}$$ (37)

Eqs (36g) and (36h) are two different discretizations of Eq (7b), and they will be used in different stages of the implementation as discussed below.

Rewrite Eq (36a) into

$$\begin{array}{c} \nabla^{2}\left[\nabla^{2}\phi^{n+1}-\frac{S}{\eta^{2}}\phi^{n+1}\right]+\frac{\gamma_{0}}{\lambda \gamma_{1}\Delta t}\phi^{n+1}=Q=Q_{1}+\nabla^{2}Q_{2}, \end{array}$$ (38)

where $Q_{1}=\frac{1}{\lambda \gamma_{1}}[g^{n+1}-{\tilde{u}}^{*,n+1}\cdot \nabla \phi^{*,n+1}+\frac{\hat{\phi }}{\Delta t}],$ and $Q_{2}=-\frac{S}{\eta^{2}}\phi^{*,n+1}+h\left(\phi^{*,n+1}\right).$ Eq (38) can be reformulated into an equivalent form (see [13, 34])

$$\begin{array}{cc} & \nabla^{2}\psi^{n+1}-\left(\alpha +\frac{S}{\eta^{2}}\right)\psi^{n+1}=Q, \end{array}$$ (39a)

$$\begin{array}{c} \nabla^{2}\phi^{n+1}+\alpha \phi^{n+1}=\psi^{n+1}, \end{array}$$ (39b)

where *ψ*^*n*+1^ is an auxiliary phase field function, and the constant *α* is given by $\alpha =-\frac{S}{2\eta^{2}}[1-\sqrt{1-\frac{4\gamma_{0}}{\lambda \gamma_{1}\Delta t}{\left(\frac{\eta^{2}}{S}\right)}^{2}}].$

In light of Eq (39b), we can transform Eq (36c) into

$$\begin{array}{c} \psi^{n+1}=\alpha \phi_{b}^{n+1}+h\left(\phi_{b}^{n+1}\right)-g_{b}^{n+1},\;\text{on}\;\partial \Omega_{i}. \end{array}$$ (40)

Similarly, Eq (36d) is transformed into

$$\begin{array}{c} n\cdot \nabla \psi^{n+1}=n\cdot \nabla Q_{2}+\left(\alpha +\frac{S}{\eta^{2}}\right)\left\{\frac{3\sigma }{4\lambda }\cos\theta_{s}\left[1-{\left(\phi^{*,n+1}\right)}^{2}\right]+g_{c2}^{n+1}\right\}\\ +g_{c1}^{n+1},\;\text{on}\;\partial \Omega_{w}. \end{array}$$ (41)

Eq (36f) is transformed into

$$\begin{array}{c} n\cdot \nabla \psi^{n+1}=n\cdot \nabla Q_{2}+\left(\alpha +\frac{S}{\eta^{2}}\right)n\cdot \nabla \phi^{n+1}+g_{a1}^{n+1},\;\text{on}\;\partial \Omega_{o}. \end{array}$$ (42)

We next derive the weak forms for the Eqs (39a) and (39b) in order to facilitate the implementation with *C*^0^ spectral elements. Let $H_{\phi 0}^{1}\left(\Omega \right)=\{\;v\in H^{1}{\left(\Omega \right)\;:\;v|}_{\partial \Omega_{i}}=0\;\},$ and $\varpi \in H_{\phi 0}^{1}\left(\Omega \right)$ denote the test function. Taking the *L*^2^ inner product between ϖ and Eq (39a) and integrating by part, we get the weak form about *ψ*^*n*+1^,

$$\begin{array}{c} \begin{array}{cc} & \int_{\Omega }\nabla \psi^{n+1}\cdot \nabla \varpi +\left(\alpha +\frac{S}{\eta^{2}}\right)\int_{\Omega }\psi^{n+1}\varpi =-\int_{\Omega }Q_{1}\varpi +\int_{\Omega }\nabla Q_{2}\cdot \nabla \varpi \\ & +\left(\alpha +\frac{S}{\eta^{2}}\right)\int_{\partial \Omega_{w}}\left\{\frac{3\sigma }{4\lambda }\cos\theta_{s}\left[1-{\left(\phi^{*,n+1}\right)}^{2}\right]+g_{c2}^{n+1}\right\}\varpi +\int_{\partial \Omega_{w}}g_{c1}^{n+1}\varpi \\ & +\left(\alpha +\frac{S}{\eta^{2}}\right)\int_{\partial \Omega_{o}}\left(-D_{0}{\left.\frac{\partial \phi }{\partial t}\right|}^{*,n+1}+g_{a2}^{n+1}\right)\varpi +\int_{\partial \Omega_{o}}g_{a1}^{n+1}\varpi ,\;\forall \varpi \in H_{\phi 0}^{1}\left(\Omega \right), \end{array} \end{array}$$ (43)

where we have used Eqs (41), (42) and (36g). Note that ${\frac{\partial \varphi }{\partial t}|}^{*,n+1}$ is given by Eq (37).

Again let $\varpi \in H_{\phi 0}^{1}\left(\Omega \right)$ denote the test function. Taking the *L*^2^ inner product between ϖ and Eq (39b) and integrating by part, we obtain the weak form about *ϕ*^*n*+1^,

$$\begin{array}{c} \begin{array}{cc} & \int_{\Omega }\nabla \phi^{n+1}\cdot \nabla \varpi -\alpha \int_{\Omega }\phi^{n+1}\varpi +\frac{\gamma_{0}D_{0}}{\Delta t}\int_{\partial \Omega_{o}}\phi^{n+1}\varpi \\ & =-\int_{\Omega }\psi^{n+1}\varpi +\int_{\partial \Omega_{o}}\left(\frac{D_{0}}{\Delta t}\hat{\phi }+g_{a2}^{n+1}\right)\varpi \\ & +\int_{\partial \Omega_{w}}\left\{\frac{3\sigma }{4\lambda }\cos\theta_{s}\left[1-{\left(\phi^{*,n+1}\right)}^{2}\right]+g_{c2}^{n+1}\right\}\varpi ,\;\forall \varpi \in H_{\phi 0}^{1}\left(\Omega \right), \end{array} \end{array}$$ (44)

where we have used Eqs (36e) and (36h).

Eqs (43) and (44) are in weak forms, and all the terms involved therein can be computed directly using *C*^0^ elements. These equations can be discretized in space using *C*^0^ spectral elements in the standard fashion. Note that these two equations are de-coupled.

In summary, given $\left({\tilde{u}}^{n},\phi^{n}\right)$, our final algorithm consists of the following procedure, which we refer to as **AdvancePhase**. It produces (*ψ*^*n*+1^, *ϕ*^*n*+1^,∇^2^ *ϕ*^*n*+1^) as follows

**AdvancePhase procedure:**
- Solve Eq (43), together with the Dirichlet condition Eq (40) on ∂Ω_*i*_, for *ψ*^*n*+1^;
- Solve Eq (44), together with the Dirichlet condition Eq (36b) on ∂Ω_*i*_, for *ϕ*^*n*+1^;
- Compute ∇^2^ *ϕ*^*n*+1^ according to Eq (39b) by ∇^2^ *ϕ*^*n*+1^ = *ψ*^*n*+1^−*αϕ*^*n*+1^.
